# Identification of genes specifically or preferentially expressed in maize silk reveals similarity and diversity in transcript abundance of different dry stigmas

**DOI:** 10.1186/1471-2164-13-294

**Published:** 2012-07-02

**Authors:** Xiao Hui Xu, Hao Chen, Ya Lin Sang, Fang Wang, Jun Ping Ma, Xin-Qi Gao, Xian Sheng Zhang

**Affiliations:** 1 State Key Laboratory of Crop Biology, Shandong Key Laboratory of Crop Biology, College of Life Sciences, Shandong Agricultural University, Tai’an, Shandong, China; 2 College of Forestry, Shandong Agricultural University, Tai’an, Shandong, China

## Abstract

**Background:**

In plants, pollination is a critical step in reproduction. During pollination, constant communication between male pollen and the female stigma is required for pollen adhesion, germination, and tube growth. The detailed mechanisms of stigma-mediated reproductive processes, however, remain largely unknown. Maize (*Zea mays* L.), one of the world’s most important crops, has been extensively used as a model species to study molecular mechanisms of pollen and stigma interaction. A comprehensive analysis of maize silk transcriptome may provide valuable information for investigating stigma functionality. A comparative analysis of expression profiles between maize silk and dry stigmas of other species might reveal conserved and diverse mechanisms that underlie stigma-mediated reproductive processes in various plant species.

**Results:**

Transcript abundance profiles of mature silk, mature pollen, mature ovary, and seedling were investigated using RNA-seq. By comparing the transcriptomes of these tissues, we identified 1,427 genes specifically or preferentially expressed in maize silk. Bioinformatic analyses of these genes revealed many genes with known functions in plant reproduction as well as novel candidate genes that encode amino acid transporters, peptide and oligopeptide transporters, and cysteine-rich receptor-like kinases. In addition, comparison of gene sets specifically or preferentially expressed in stigmas of maize, rice (*Oryza sativa* L.), and Arabidopsis (*Arabidopsis thaliana* [L.] Heynh.) identified a number of homologous genes involved either in pollen adhesion, hydration, and germination or in initial growth and penetration of pollen tubes into the stigma surface. The comparison also indicated that maize shares a more similar profile and larger number of conserved genes with rice than with Arabidopsis, and that amino acid and lipid transport-related genes are distinctively overrepresented in maize.

**Conclusions:**

Many of the novel genes uncovered in this study are potentially involved in stigma-mediated reproductive processes, including genes encoding amino acid transporters, peptide and oligopeptide transporters, and cysteine-rich receptor-like kinases. The data also suggest that dry stigmas share similar mechanisms at early stages of pollen-stigma interaction. Compared with Arabidopsis, maize and rice appear to have more conserved functional mechanisms. Genes involved in amino acid and lipid transport may be responsible for mechanisms in the reproductive process that are unique to maize silk.

## Background

In angiosperms, stigmas discriminate between compatible and incompatible pollen grains by preventing the adhesion, hydration, penetration, and growth of incompatible pollen [1]. After landing on the stigma, compatible pollen grains hydrate and germinate to produce pollen tubes that penetrate the stigma surface. These pollen tubes then utilize signals and nutrients from the stigma or style for successful pollination and fertilization. To carry out these reproductive processes, two types of stigmas have developed in flowers. One type is the wet stigma, which deposits fluid secretions or exudates on its surface. The other is the dry stigma, which has an intact papillate surface on which the stigma interacts directly with pollen grains [2-4]. Species with dry stigmas include the model plant species Arabidopsis (*Arabidopsis thaliana* [L.] Heynh.) and the economically important grasses rice (*Oryza sativa* L.) and maize (*Zea mays* L.). With the exception of self-incompatibility (SI), relatively little is known about the molecular mechanisms of plant reproduction in dry stigmas.

Because many genetic, cellular, and biotechnological tools, as well as a maize reference genome (inbred line B73), are currently available, maize has been proposed as a model grass to study the function of the dry stigma in reproduction [5]. Maize silk is a specialized elongated tissue that is functionally equivalent to the stigma and style of a typical pistil [6,7]. Hairs that are located on the silk surface serve as receptive structures to support pollen adhesion, hydration, and germination. To possess this unique function, maize silk must express genes and produce proteins required for successful pollination, and many of these genes might be specifically or preferentially expressed in silk. A genome-wide identification of silk-specific/preferential genes might therefore serve as an initial step in the analysis of the molecular pathways involved in reproduction. To obtain silk-specific/preferential genes at the whole genome level, we performed a deep sequencing analysis in maize silk using next-generation sequencing [8] and microarray analysis.

Several mechanisms related to pollination and fertilization has been implicated in pollen germination, pollen tube guidance, and pollen-ovule interactions [9,10]. In contrast, little is understood about the molecular mechanisms of stigmatic reproductive functions. A major challenge to the study of reproductive functions of the stigma is the dearth of informative mutants, especially for grasses [4]. Exploring stigma function through comparative genomics is likely to yield valuable information and build a link between morphological and genetic analyses. Candidate genes thus identified can be selected for further reverse genetic analysis. To date, genome-wide transcriptome analysis of dry stigmas has been conducted in Arabidopsis, rice, and maize [11-15]. The Arabidopsis stigma dataset was established by cDNA subtraction and microarray analysis of stigma tissues [11,12]. In rice, the stigma dataset was generated using a 57 K Affymetrix rice whole genome array and 10 K cDNA microarray [13]. Microarray and RNA-seq analyses have recently been performed on different maize tissues of inbred line B73 at vegetative and reproductive stages and on silk at the anthesis stage [14,15].

In this study, RNA-seq was performed to analyze transcript abundance profiles of mature silk (MS), mature pollen (MP), mature ovary (MO), and 6-day-old seedling (SL). To build a profile of candidate silk genes that are involved in early pollination events, genes expressed in maize silk were compared with those expressed in the other three tissues. We identified 1,427 genes that were specifically or preferentially expressed in maize silk. Bioinformatic analysis of the maize silk dataset not only identified pathways and genes that have been shown to be essential for pollination, but also a number of genes with novel or previously-unidentified functions, such as those encoding amino acid transporters, peptide and oligopeptide transporters, and cysteine-rich receptor-like kinases (CRKs). In addition, we characterized conserved and distinct features of dry stigmas by comparing stigma-specific/preferential datasets of Arabidopsis, rice, and maize.

## Results and discussion

### Transcriptome analysis of reproductive organs and seedlings in maize

**Figure 1:**
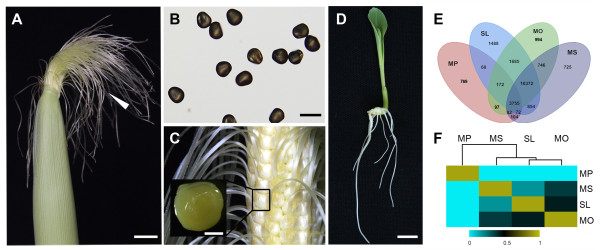
**Distribution of genes expressed in four studied tissues and correlation matrix of their RNA-seq libraries.** (**A**) Mature silk (MS) (arrowhead). (**B**) Mature pollen (MP). (**C**) Mature ovaries (MO) (rectangle). (**D**) Six-day-old seedling (SL). (**E**) A four-way Venn diagram of genes expressed in MP, MS, MO, and SL. The number of genes expressed in each tissue is circled. (**F**) Correlation matrix of RNA-seq libraries for four maize tissues. Scale bars = 1 cm (**A**, **D**), 100 μm (**B**), and 1 mm (**C**).

To identify genes involved in reproductive processes, an RNA-seq analysis was performed on MS that emerged from the husk over 3 days (Figure 1A). To eliminate transcripts involved in vegetative growth and basic metabolism in silk tissue, the transcriptomes of MP (Figure 1B), MO (Figure 1C), and SL (Figure 1D) were also sequenced. This enabled us to distinguish transcripts specific to silk from transcripts that contribute to common plant functions. mRNAs from the four tissues were used to construct libraries, which were then sequenced on an Illumina HiSeq 2000 system. After sequencing quality control (Additional file 1) and removal of the “dirty” raw reads (see Methods), the number of purity-filtered reads ranged from 6,145,170 to 6,764,608 per library (Additional file 2).

To identify genes corresponding to reads in each library, the purity-filtered reads were mapped to version 2 of the maize B73 reference genome (AGPv2) [16] with the Short Oligonucleotide Alignment Program 2 (SOAP2) aligner (Additional files 2 and 3) [17]. To make the libraries meaningful, reads that appeared less than four times per million unique mapped reads were eliminated from further statistical analysis. As a result, a total of 16,710 genes were expressed in MS, 5,119 in MP, 17,903 in MO, and 18,466 in SL (Figure 1E, Additional file 4). As shown in Figure 1E, the extent of gene overlap was determined by comparing the transcript libraries of the four experimental tissues. Many genes (62.07%) expressed in MS were also detected in MO and SL but not in MP, implying that the transcriptome of MS is closer to MO and SL than to MP.

To investigate the difference between the transcriptome profile of MS and those of MP, MO, and SL, a Pearson correlation coefficient (PCC) analysis was performed on the sequencing libraries derived from the four tissues (Figure 1F). Overall, PCC values ranged from 0.0093 to 0.49 (Figure 1F, Additional file 5). As expected, SL tissue was poorly correlated with MP (PCC = 0.012) and MS (PCC = 0.23). Interestingly, however, MS showed a higher correlation with MO (PCC = 0.38) than with MP and SL. The results indicate that the expression patterns of genes in MS were similar to those in MO, in which many genes may be involved in female reproduction. To further clarify the functional difference between the four tissue transcriptomes, a comparative gene ontology (GO) analysis (Parametric Analysis of Gene Set Enrichment, PAGE) [18] was performed on the genes expressed in the four tissues (Additional file 6). Notably, the MS-expressed genes were most represented in biological processes such as cell wall function, lipid transport, carbohydrate catabolism, and aminoglycan metabolism. Interestingly, genes relating to neuropeptides and neurotransmitters were overrepresented in MS, while genes involved with these functions were barely detected in the other three tissues. Taken together, the results show that MS-expressed genes were distinct from those expressed in vegetative tissues (SL) and other reproductive organs (MP and MO).

### Comparison between RNA-seq data and microarray and real-time PCR data

**Figure 2:**
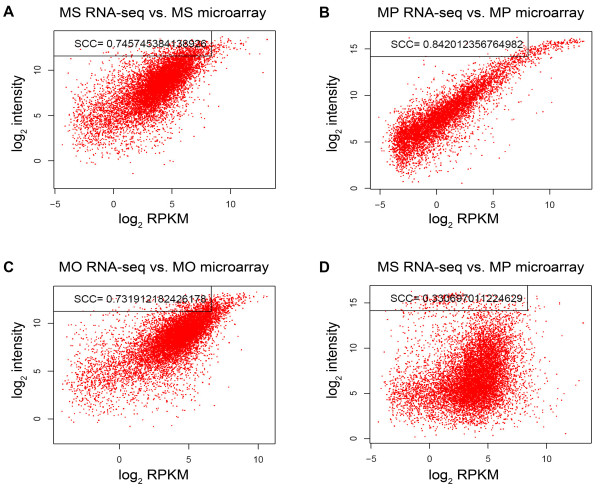
**Comparison between RNA-seq and microarray data.** Genes without splicing variants and present in both RNA-seq and microarray data sets were selected for comparison between the two platforms. Log_2_-transformed RNA-seq values (RPKM) and microarray-normalized expression values are shown as scatter plots. Spearman correlation coefficients (SCCs) for the comparison were calculated using the R package.

The transcript abundance profiles of MS, MP, and MO were analyzed using the Affymetrix 18 K maize genome array, and the results were compared with the RNA-seq data. Spearman rank correlation coefficients (SCCs) of log_2_-transformed expression values were calculated between the three corresponding tissue pairs in the two datasets. The SCC value between the dissimilar tissue pair MS and MP was calculated as a negative control (Figure 2). As a result, SCC values between the same tissues (0.73–0.84) were greater than those between dissimilar tissue pair (0.33). In addition, we used a scatter diagram to plot the log_2_-transformed expression values from the microarray and RNA-seq analyses of the three same tissue pairs and the one dissimilar tissue pair (Figure 2). These plots illustrate that similar expression patterns were detected for the same tissue using the two different techniques. Transcript abundance values generated by the RNA-seq analysis were distributed much more broadly than those from the microarray analysis. This difference may be due to the wider range of expression detected by RNA-seq compared with the microarray. Overall, these results indicate that data from RNA-seq and microarray analyses were comparable.

**Figure 3:**
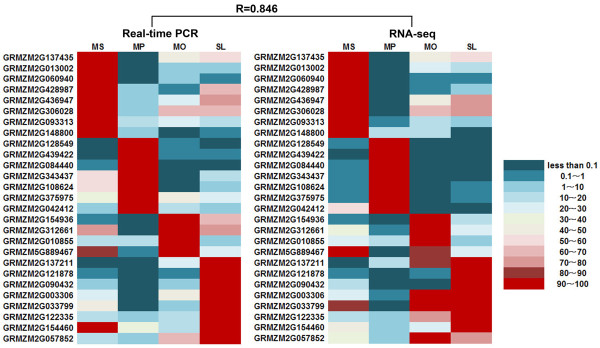
**Validation of RNA-seq results by real-time PCR.** Transcript abundance represented by the two heat maps are the average of transcript abundance values from three independent real-time PCR experiments (left) and RPKM values of the RNA-seq analysis (middle). For each gene, the tissue with the maximum transcript abundance was set to 100, and relative transcript abundances in the other three tissues were calculated based on this maximum. Relative expression is represented by color scales as indicated (right). R is the correlation coefficient value between the two platforms.

Real-time PCR analysis was employed to further validate the expression of genes examined by RNA-seq in MS, MP, MO, and SL (Figure 3). We randomly selected 27 genes, including eight with putative functions belonging to different gene families, nine encoding hypothetical proteins, five encoding expressed proteins, and five without annotation (Additional file 7). Results showed that the expression patterns of these genes were well correlated with the RNA-seq results (R = 0.846), supporting the reliability of our RNA-seq data.

### Identification and functional classification of genes specifically or preferentially expressed in maize silk

**Figure 4:**
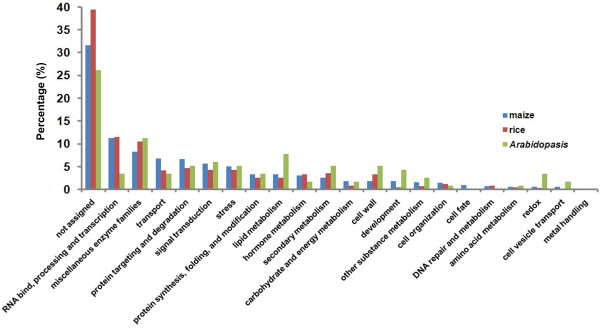
**Classification of stigma specifically/preferentially expressed genes in maize, rice, and Arabidopsis.** Classification results were obtained using the MapMan classification system.

Analysis of genes specifically or preferentially expressed in MS might lead to a better understanding of the involvement of maize silk in plant reproduction. To this end, 725 genes specific to the MS library, compared with those from the other three tissues, were identified (Figure 1E). A significance of digital gene expression analysis [19] was then conducted to compare MS vs. MP, MS vs. MO, and MS vs. SL. Using fold change ≥ 2, false discovery rate (FDR) < 1e-05, and P-value < 1e-05 as criteria for each comparison, 702 genes were identified from all three comparisons, and their transcript abundances were significantly greater in MS compared with MP, MO, and SL. These genes were considered to be preferentially expressed in MS. A total of 1,427 genes specifically or preferentially expressed in the silk were thus identified (Additional file 8A). These genes were subsequently divided into 21 categories and one unassigned gene category using MapMan [20]. The genes involved in RNA binding, processing, and transcription, protein targeting and degradation, signal transduction, miscellaneous enzyme families, and transport were well represented (Figure 4).

**Figure 5:**
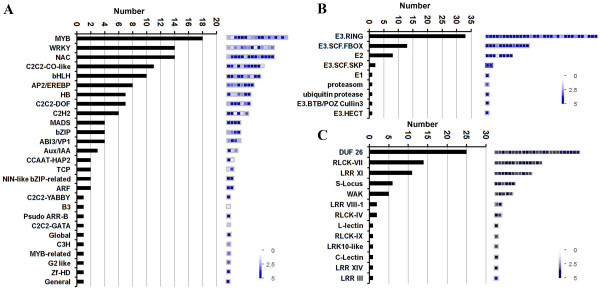
**Distribution of TF, RLK, and UPS-related genes in the MS-specific/preferential dataset.** MS specifically or preferentially expressed genes relating to (**A**) TF (transcription factor), (**B**) UPS (ubiquitin-proteasome system), and (**C**) RLK (receptor-like kinase), were identified using MapMan. The number of genes in each category is shown in the histogram (left). Color scales on the right indicate the relative transcript abundance of each gene (determined by the log_2_-transformed RPKM values) included in the given categories.

In the category of RNA binding, processing, and transcription, 128 genes encoding transcription factors (TFs) were identified. Most (58.6%) belonged to six families: MYB, WRKY, NAC, C2C2-CO-like, bHLH, and AP2-EREBP (Figure 5A, Additional file 8A). Expression and genetic analyses have substantiated the involvement of all six TF families in flower development and reproduction [21-24]. It is unclear, however, whether these TFs are directly involved in pollen-stigma interaction.

Genes encoding ubiquitin-proteasome-system (UPS) proteins constituted a large proportion (63.5%) of the protein targeting and degradation category (Figure 5B, Additional file 8A). Recent studies have shown that UPS proteins function in both sporophytic and gametophytic SI [25]. An E3 ubiquitin ligase, ARMADILLO REPEAT CONTAINING 1 (ARC1), is required for the rejection of incompatible pollen in sporophytic SI in *Brassica* pistils [26], and F-box proteins (SCF) assist degradation of S-RNase in non-self pollen tubes in gametophytic SI [27]. Although a large number of UPS-related genes were identified in maize silk, SI has not been reported in maize; gametophytic SI has been found, however, in wild relatives of maize [5]. Moreover, ubiquitin has been shown to be involved in human sperm discrimination during fertilization [28,29]. Abnormal sperm is ubiquitinated on its surface, and then phagocytosed by epididymis cells [29]. Our findings provide new insights into the function of ubiquitin during pollen-stigma interaction in plants. The roles of genes encoding UPS-related proteins in maize silk deserve further investigation.

Seventy-one genes encoding receptor-like kinases (RLKs) were identified from the signal transduction category. These genes were classified into 13 subfamilies (Figure 5C, Additional file 8A). The largest subfamily includes 26 receptor-like kinases (DUF26 RLK) or CRKs, which play important roles in regulation of pathogen defense and programmed cell death [30-32]. Despite the essential roles of cysteine-rich proteins (CRPs) in SI and in pollen tube growth and guidance, the function of CRKs in pollen-pistil interaction has not been reported. However, based on a recent study involving PDLP1 with two DUF26 domains, we tentatively propose that the enrichment of CRKs in maize silk suggests an enhanced cell-to-cell trafficking role in this species [33]. The second largest category of genes encodes cytoplasmic receptor-like kinases (RLCKs). These kinases are directly activated by calcium/calmodulin that modulates pollen tube growth and pollen-pistil interaction [34,35]. The function of stigmatic RLCKs in compatible pollination is still unknown. The third category consists of genes encoding leucine-rich repeat RLKs that are involved in regulation of pollen tube growth in petunia and tomato [36-38]. Examples in this category include LePRK1 and LePRK2, which are two well-defined pollen kinases that act in pollen-pistil interaction primarily by perceiving signals from pistil to regulate pollen tube growth. Additionally, six *S*-locus receptor kinase (SRK) proteins were identified from the MS-specific/preferential dataset. In *Brassica* and Arabidopsis, SRKs are usually localized in stigma papilla cells, and interact with *S*-locus cysteine rich proteins to control SI response [39]. We therefore suggest that genes encoding RLKs are probably important participants in stigma-mediated reproductive processes, but their functions in compatible pollination require further genetic elucidation.

### Bioinformatic analysis revealed a number of maize silk genes that may act in reproductive processes

To identify GO terms significantly enriched in genes specifically or preferentially expressed in MS, a singular enrichment analysis (SEA) [18] was performed. Genes were classified based on their cellular component, molecular function, or biological process, and 22 GO terms were overrepresented in MS based on P-value < 0.001 and FDR ≤ 0.05 cutoffs (Table 1). Most of the overrepresented GO terms were involved in transport, cell wall regulation, signaling, and lipid metabolism, which are essential in stigmatic reproductive processes [1,40]. This suggests that the identified MS-specific/preferential dataset reflects the reproductive characteristics of maize silk.

**Table 1:** Overrepresented functional GO terms of MS-specific/preferential genes

**GO Term**^**a**^	**Term type**^**a**^	**Query item**^**b**^	**Query total**^**c**^	**Bg item**^**d**^	**Bg total**^**e**^	**P-value**^**f**^	**FDR**^**g**^
amino acid transport	P	10	900	52	39203	2.80E-07	0.00021
protein amino acid phosphorylation	P	93	900	2387	39203	2.70E-06	0.00082
oligopeptide transport	P	12	900	105	39203	6.10E-06	0.0011
fatty acid metabolic process	P	14	900	187	39203	0.00013	0.014
lipid transport	P	10	900	104	39203	0.00015	0.014
cell wall macromolecule catabolic process	P	8	900	73	39203	0.00028	0.022
chitin catabolic process	P	6	900	43	39203	0.00044	0.03
drug transport	P	11	900	154	39203	0.00097	0.05
integral to membrane	C	75	900	2156	39203	0.00055	0.029
peroxisome	C	5	900	33	39203	0.0009	0.029
protein serine/threonine kinase activity	F	92	900	2248	39203	4.40E-07	0.00013
monooxygenase activity	F	33	900	553	39203	1.30E-06	0.00029
heme binding	F	43	900	876	39203	6.20E-06	0.0007
acyltransferase activity	F	16	900	187	39203	8.80E-06	0.0008
amino acid transmembrane transporter activity	F	6	900	28	39203	3.60E-05	0.0024
electron carrier activity	F	42	900	914	39203	3.50E-05	0.0024
transferase activity, transferring hexosyl groups	F	28	900	542	39203	9.40E-05	0.0053
sugar binding	F	12	900	150	39203	0.00021	0.0095
secondary active transmembrane transporter activity	F	22	900	421	39203	0.00042	0.018
chitinase activity	F	6	900	43	39203	0.00044	0.018
lipid binding	F	13	900	202	39203	0.00093	0.035
drug transmembrane transporter activity	F	11	900	154	39203	0.00097	0.035

^a^GO term classifications: P, Biological Process; C, Cellular Component; F, Molecular Function.

^b^Query item number in MS preferential expressed genes.

^c^Total annotated query item number in agriGO.

^d^Query item number in maize genome version 5a.

^e^Total annotated item number in maize genome version 5a.

^f^Determined by Fisher exact test.

^g^Determined by Benjamini–Hochberg–Yekutieli procedure.

GO terms with P-value < 0.001 and FDR ≤ 0.05 were regarded as overrepresented terms.

Based on GO cellular components, the two GO terms designated as integral to membrane and peroxisome were overrepresented in the MS-specific/preferential datasets (Table 1). Several studies have shown that proteins integral to membranes play important roles in pollination [1]. On the other hand, the functions of all five peroxisome-associated proteins are unknown (Table 1). We therefore identified their homologous genes in Arabidopsis by performing a BlastP search against the Arabidopsis protein database [41]. Two identified genes encode peroxins, two gene products may be related to peroxisome biogenesis, and one gene product is involved in fatty acid metabolism. Among these genes, peroxin is essential for female and male gametophytic recognition, according to an analysis of the loss-function mutant *abstinence by mutual consent*[42].

Based on the GO molecular function and biological process analyses, terms with lipid-related annotation were overrepresented, including lipid binding, lipid transport, and fatty acid metabolic processes. This is consistent with previous findings that lipids play important roles in pollination [43-45]. We therefore further analyzed lipid metabolism-related genes in the MS-specific/preferential gene dataset using MapMan [20] (Additional file 8A). Nearly 30% of the genes were involved in fatty acid synthesis and elongation. These overrepresented genes encode members of 3-ketoacyl-COA synthase (KCS), long-chain acyl-CoA synthase, acyl-activating enzyme, and acyl-CoA binding protein families. KCS proteins are responsible for the synthesis of very-long-chain fatty acids (VLCFAs), which are major components of the cuticle (wax and cutin) covering the stigma [46]. In Arabidopsis, mutations in *FIDDLEHEAD* and *CER* genes have been shown to disrupt VLCFA biosynthesis, and thus affect pollen-stigma interaction, indicating important roles for VLCFAs in reproductive processes [45,47-49]. We also identified a number of genes involved in phospholipid synthesis in the MS-specific/preferential gene dataset. Phospholipids regulate pollen germination and tube growth [36,50], but their functions in the stigma are unknown. Moreover, genes involved in lipid transport and degradation were also overrepresented. Most of the genes encoding lipid binding proteins and lipid transfer proteins (LTPs) that have been shown to be essential to successful pollen adhesion and tube guidance were studied in wet stigma species [51-53]. In addition, we found many genes involved in lipid degradation and desaturation, such as genes encoding triacylglycerol lipase, acyl-CoA thioesterase, phospholipase D, GDSL lipases, and fatty acid desaturase. According to several recent studies [1], some of these proteins may support pollen hydration and germination. For example, EXL4, a member of the GDSL family, is involved in rapid initiation of pollen hydration on the stigma [54]. Because application of unsaturated triacylglycerols to stigma-less tobacco plants promotes pollen hydration and germination [43], fatty acid desaturase might be required for reproductive success in the stigma.

**Figure 6:**
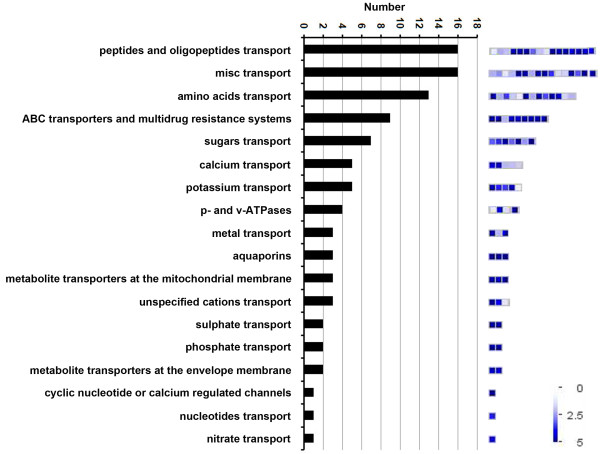
**Distribution of transport-related genes in the MS-specific/preferential gene set.** MS-specific/preferential genes relating to transport function were identified using MapMan. The number of genes in each category is shown in the histogram (left). Their relative transcript abundance determined by the log_2_-transformed RPKM values in MS is indicated by color scales (right).

Genes involved in various transport processes, including transport of amino acids, oligopeptides, lipids, and drugs, were overrepresented based on GO biological process analysis (Table 1). The stigma is a specialized tissue that secretes materials supporting the growth of compatible pollen tubes and inhibiting the growth of incompatible pollens [55]. To investigate transport-related genes in the MS preferential gene dataset in detail, a MapMan functional analysis [20] was performed (Figure 6, Additional file 8A). Unexpectedly, genes encoding amino acid, peptide, and oligopeptide transporters constituted a large proportion of the transport-related genes. Results from a recent study showing that amino acids transported from the pistil regulate glutamate receptor-like proteins, which in turn control pollen tube Ca^2+^ concentration and growth, provide an insight into amino acid transporter function in maize silk [56]. Furthermore, *AtPTR5*, a gene encoding a peptide transporter in Arabidopsis, was found to facilitate peptide uptake into germinating pollen [57]. Another peptide transporter gene, *AtPTR1*, is also expressed in the stigma and style, and may function in a similar manner to supply peptides for initial pollen tube growth [58]. In addition, as shown in Figure 6, genes for potassium, unspecific cation, divalent, gated, and calcium channel proteins were also well represented. Among these, potassium and calcium channel proteins were overrepresented. In the stigma, Ca^2+^ is required for pollen germination and pollen tube guidance [59,60]. Although potassium channel proteins and K^+^ have been implicated in regulation of pollen tube growth [61], their roles in the pistil are yet to be investigated.

Approximately 14% of genes identified in the maize genome encode proteins with putative N-terminal signal peptides, and nearly 19% of the genes specifically or preferentially expressed in MS encode proteins involved in the secretory pathway (Additional file 9). The genes enriched in maize silk are likely participants in extracellular signaling. Various secreted polypeptides have been reported to be involved in signaling during pollen-pistil interaction. These include pollen tube attractants, factors for pollen germination and tube growth, and determinants of SI. Interestingly, most of these are CRPs [62], but so far, no CRPs have been identified from stigmas in the Poaceae. In this study, we used sequence motif models generated from each CRP subfamily [63] to identify CRPs in the MS preferential datasets (see methods and Additional file 10). Identified CRP sequences have N-terminal signal peptides, and CRP subcellular locations include the cell wall, plasma membrane, and endomembrane system (Additional file 10). Classes of CRPs identified in the maize silk preferential dataset include LTPs, lipid binding proteins, and serine-type endopeptidase inhibitors. Style/stigma cysteine-rich protein, a plant LTP, is involved in adhesion of the pollen tube to the transmitting tract [51-53]. The large number of LTPs identified from maize silk may provide an explanation for how pollen tubes can adhere to the epidermal surface of the transmitting tract during their long journey through the maize silk. Lipid binding proteins are also involved in pollen-pistil interactions. For example, glycine-rich proteins with lipid binding domains reduce pollen hydration rates [64]. Although serine-type endopeptidase inhibitors have not been implicated in pollen-pistil interactions, these inhibitors are known to participate in cell-to-cell communication during plant-pathogen interactions [65].

### Dry stigmas may share similar mechanisms in the early stages of pollen-stigma interaction

Dry stigma preferential datasets have currently been generated for two species: Arabidopsis and rice [12,13]. To reveal the conservation or divergence of stigma preferential genes among different dry stigmas, we compared gene sequences generated from these earlier studies with our MS-specific/preferential gene sets.

For this purpose, protein sequences of 1,427 maize silk-specific/preferential genes were extracted and blasted against the MSU rice protein database [66] and Arabidopsis protein database [41], with homologous proteins filtered using an E-value ≤ 1e-10 cutoff. We then selected homologous protein-encoding genes that were also included in rice and Arabidopsis stigma-specific/preferential datasets. For each maize gene, only the best hit (lowest homologous protein E-value) of these genes in the rice or Arabidopsis stigma-specific/preferential datasets was selected as the homologous gene. We found that 471 genes specifically or preferentially expressed in maize silk (32.96% of the MS-specific/preferential dataset) were homologous to 213 rice stigma-specific/preferential genes (38.87% of the rice stigma-specific/preferential dataset) (Additional file 11A), while 140 maize silk-specific/preferential genes (9.8% of the MS-specific/preferential dataset) were homologous to 37 Arabidopsis stigma-specific/preferential genes (32.17% of the Arabidopsis stigma-specific/preferential dataset) (Additional file 11B). We also blasted the protein sequences of the 548 rice and 115 Arabidopsis stigma-specific/preferential genes against the maize AGPv2 5b filtered gene set peptide database [16]. Homologous maize genes were identified using the same criterion as above. As a result, 221 rice stigma-specific/preferential genes (40.32% of the rice stigma-specific/preferential dataset) matched 179 maize silk-specific/preferential genes (12.54% of the MS-specific/preferential dataset) (Additional file 11C), and 53 Arabidopsis stigma-specific/preferential genes (46.09% of the Arabidopsis stigma-specific/preferential dataset) matched 48 MS-specific/preferential genes (3.77% of the MS-specific/preferential dataset) (Additional file 11D). From these BLAST results, we found that the three dry stigma-specific/preferential datasets match each other well, suggesting conserved mechanisms across monocots and dicots.

We found that 107 maize silk-specific/preferential genes had homologous matches in both rice (45 genes) and Arabidopsis (23 genes) stigma-specific/preferential datasets (Additional file 12A). The proteins encoded by some of these genes (17 out of 107) have been shown to be involved in the early events of pollen-stigma interaction. For example, *S*-locus glycoproteins participate in pollen adhesion [67], aquaporins and GDSL lipases regulate the process of pollen hydration [54,68], fatty acid-elongation-related proteins participate in pollen hydration and pollen tube germination [43,44], and expansins and pectinesterases function in initial growth and penetration of the pollen tube into the stigma surface [69-71]. Despite differences in their morphology and transcript abundance patterns, dry stigmas of maize, rice, and Arabidopsis may share similar mechanisms in the early stages of stigma-mediated reproductive processes.

### Maize stigma shares more conserved functional mechanisms with rice than with Arabidopsis

To compare the characteristics of stigma-specific/preferential genes among maize, rice, and Arabidopsis, we classified the genes in all three dry stigma datasets into 21 categories and one unassigned gene category using MapMan [20] analysis, and compared their functional profiles (Figure 4). Functional distributions of the genes in maize and rice were very similar to each other, but differed from those of Arabidopsis. Moreover, genes related to transcription and hormone functions constituted relatively larger proportions in maize and rice than in Arabidopsis. These results suggest that transcriptional regulation networks and hormone-mediated regulation play more prominent roles in stigmas of maize and rice than in those of Arabidopsis.

The expression profile of maize silk-specific/preferential genes shared more similarity with that of rice than with that of Arabidopsis (Additional file 12B). A large number of genes were conserved between maize (364) and rice (185) stigma-specific/preferential datasets, but the same genes were not present in the Arabidopsis dataset. To further analyze common characteristics shared between maize and rice, we performed GO analysis on these conserved genes [18]. Gene terms related to protein serine/threonine kinase activity, protein amino acid phosphorylation, and oligopeptide transport were overrepresented (Table 2). Because maize and rice are both members of Poaceae while Arabidopsis belongs to Brassicaceae, we suggest that mechanisms related to stigma function are more similar in species with closer genetic backgrounds.

**Table 2:** Overrepresented GO terms of MS-specific/preferential genes that only hit rice stigma dataset

**GO Term**^**a**^	**Term type**^**a**^	**Query item**^**b**^	**Query total**^**c**^	**Bg item**^**d**^	**Bg total**^**e**^	**P-value**^**f**^	**FDR**^**g**^
protein amino acid phosphorylation	P	67	294	2387	39203	1.50E-17	8.60E-15
oligopeptide transport	P	12	294	105	39203	4.10E-11	2.90E-09
fatty acid biosynthesis process	P	10	294	159	39203	5.00E-07	2.60E-05
apoptosis	P	8	294	168	39203	5.00E-05	0.002
regulation of transcription	P	46	294	3352	39203	0.00024	0.0069
multidrug transport	P	5	294	79	39203	0.00035	0.0076
protein serine/threonine kinase activity	F	67	294	2248	39203	8.30E-19	2.80E-16
ATP binding	F	85	294	5133	39203	1.50E-09	6.30E-08
acyltransferase activity	F	11	294	187	39203	2.70E-07	5.70E-06
sugar binding	F	10	294	150	39203	2.90E-07	5.80E-06
transcription factor activity	F	28	294	1390	39203	7.50E-06	0.00012
sequence-specific DNA binding	F	22	294	985	39203	1.40E-05	0.00022
transporter activity	F	37	294	2411	39203	0.00011	0.0015

^a^GO term classifications: P, Biological Process; C, Cellular Component; F, Molecular Function.

^b^Query item number in MS preferential expressed genes.

^c^Total annotated query item number in agriGO.

^d^Query item number in maize genome version 5a.

^e^Total annotated item number in maize genome version 5a.

^f^Determined by Fisher exact test.

^g^Determined by Benjamini–Hochberg–Yekutieli procedure.

GO terms with P-value < 0.001 and FDR ≤ 0.05 were regarded as overrepresented terms.

### Amino acid and lipid transport-related genes are involved in distinctive mechanisms of maize silk-mediated reproductive processes

Comparison of functional profiles of the three different dry stigmas (Figure 4) revealed that genes involved in transport and in protein targeting and degradation represented a larger percentage of the MS-specific/preferential dataset than those of rice and Arabidopsis. In addition, we identified 923 genes that were distinctively represented in MS-specific/preferential dataset (Additional file 12C). GO analysis of these genes indicated that genes involved in amino acid transmembrane transporter activity and in amino acid and lipid transport were overrepresented (Table 3). A previous study has shown that nutrients in pollen can support only about 2 cm tube growth in maize silk [7]. For successful fertilization, maize silk must provide enough nutrients and appropriate signals to support pollen tube growth and guidance for a much longer distance. These supporting activities are apparently carried out by the distinctively-enriched transporters in maize silk. In accordance with this idea, we identified 13 amino acid transporter genes in the maize silk-specific/preferential dataset, but only one gene in rice and none in Arabidopsis. As shown in previous studies [56], amino acid-mediated communication is required for pollen-pistil interaction. The transporters may also transfer amino acids into the pollen tube to regulate its growth. In addition, the lipid transport-related genes uniquely represented in the maize silk-specific/preferential dataset included a number of genes encoding LTPs that may function in the adhesion of pollen tubes to the surface of transmitting tract during their growth in the silk. We therefore suggest that the distinctive expression of genes encoding amino acid and lipid transporters is consistent with the extended length of maize silk.

**Table 3:** Overrepresented GO terms of genes distinctively presented in specific/preferential dataset of maize silk

**GO Term**^**a**^	**Term type**^**a**^	**Query item**^**b**^	**Query total**^**c**^	**Bg item**^**d**^	**Bg total**^**e**^	**P-value**^**f**^	**FDR**^**g**^
lipid transport	P	9	476	104	39203	6.00E-06	0.0034
amino acid transport	P	6	476	52	39203	4.20E-05	0.0079
chitin catabolic process	P	5	476	43	39203	0.00018	0.025
tetracycline transport	P	6	476	75	39203	0.00033	0.029
response to antibiotic	P	6	476	84	39203	0.0006	0.038
amino acid transmembrane transporter activity	F	6	476	28	39203	1.00E-06	0.00019
chitinase activity	F	5	476	43	39203	0.00018	0.022
small conjugating protein ligase activity	F	13	476	324	39203	0.00023	0.022
tetracycline transporter activity	F	6	476	75	39203	0.00033	0.022
lipid binding	F	9	476	202	39203	0.00096	0.049
coenzyme binding	F	20	476	734	39203	0.001	0.049
integral to membrane	C	51	476	2156	39203	1.90E-05	0.0016
peroxisome	C	5	476	33	39203	4.80E-05	0.0016

^a^GO term classifications: P, Biological Process; C, Cellular Component; F, Molecular Function.

^b^Query item number in MS preferential expressed genes.

^c^Total annotated query item number in agriGO.

^d^Query item number in AGPv2 5a.

^e^Total annotated item number in AGPv2 5a.

^f^Determined by Fisher exact test.

^g^Determined by Benjamini–Hochberg–Yekutieli procedure.

GO terms with P-value < 0.001 and FDR ≤ 0.05 were regarded as overrepresented terms.

### A number of genes showed presence/absence variations and different expression patterns between different maize inbred lines

Two recent transcriptomic studies on maize inbred line B73 have revealed important information about temporal and spatial gene expression patterns in different tissues, and highlighted the utility of microarray and RNA-seq methods in the generation of databases for gene discovery and functional characterization [14,15].

In one of the studies, RNA-seq analysis generated transcriptome profiles of different reproductive tissues, including cobs and pollen, as well as developing seeds and leaves [15]. To make a meaningful comparison between inbred lines B73 and Zheng58, genes exclusively or preferentially expressed in silk compared with those in post-emergence cob, pollen, and leaf were extracted from the previously-reported data [15], and were searched against our MS-specific/preferential dataset. A total of 72 specific and two preferential genes in our dataset were found to be specific to previous data, and 154 specific and 153 preferential genes were preferentially expressed in the tissues of the previous studies [15] (Additional file 13A). On the other hand, 24 out of 138 silk-exclusive genes of inbred line B73 were absent from our RNA-seq dataset, and 25 of our 1,427 MS-specific/preferential genes were absent from the data derived from inbred line B73. Presence/absence variations (PAVs) between inbred lines B73 and Zheng58 have been found for a number of genes [72]. Our results indicate that besides the previously-reported PAVs, many genes show different expression patterns between these two inbred lines.

We also compared our MS-specific/preferential dataset to datasets derived from tissues absent in our study but present in the two previous studies. A comparison between our MS-specific/preferential dataset and those of leaves, cobs, ovule, tassels, anthers, pollen, seeds, embryo, and endosperm from the previously-reported RNA-seq data revealed 23 genes specifically and 166 genes preferentially expressed in silk (Additional file 13B) [15]. A comparison of our MS-specific/preferential dataset with those of roots, whole seedling, internodes, cobs, tassels, anthers, leaves, endosperms, embryos, and pericarp from the previously-reported microarray data identified nine specific and 27 preferential genes (Additional file 13C) [14]. In addition, 456 silk-specific/preferential genes in our dataset were absent from the previously-reported microarray data, which may be due to PAVs between different inbred lines or different gene expression detection ranges in RNA-seq and microarray techniques. Overall, the comparison between our MS-specific/preferential dataset and previous studies on inbred line B73 indicate there are many genes with PAVs and differential expression patterns. In addition, consistent with previous findings, these results also suggest that some genes involved in reproductive processes are also expressed in vegetative organs where they carry out similar molecular functions [73-78].

## Conclusions

In this study, 1,427 genes expressed predominantly or specifically were identified in maize silk using RNA-seq. A number of novel gene sets might be involved in stigma-mediated reproductive processes. A comparative analysis of the stigma-specific/preferential gene datasets of Arabidopsis, rice, and maize suggest that 1) different dry stigmas share similar mechanisms in the early stages of pollen-stigma interaction; 2) maize shares more conserved functional mechanisms with rice than with Arabidopsis; and 3) genes involved in amino acid and lipid transport might be responsible for the distinct functions of reproductive processes in maize silk. Further functional analysis of the identified genes will aid our understanding of stigmatic mechanisms taking place during reproduction.

## Methods

### Plant materials, growing conditions, and RNA extraction

Maize inbred line Zheng58 was used for RNA-seq and microarray analysis. For SL materials, seeds of Zheng58 were surface-sterilized with 3% sodium hypochlorite for 10 min and rinsed well with distilled water. Sterilized seeds were pre-germinated on moistened pledgets in a plant growth chamber at 25°C under a 16/8 h light/dark cycle for 6 days. The whole seedling, include both the aerial part and root, was harvested. For reproductive tissues such as MS, MP, and MO required for RNA-seq and real-time PCR, plants were grown in silt loam soil at the Shandong Agricultural University Experimental Station in Tai’an, China (35°58′ N, 116°38′ E, 150 m above sea level). Before tassels began shedding pollen and silk started growing from husk, we bagged tassels and husks of experimental plants to prevent cross-pollination from other plants and pathogen invasion. Two to 5 days after anther dehiscence, MP grains were collected in 1.5 mL Eppendorf tubes between 8:00 and 10:00 am. To ensure the collection of highly-viable MP, we shook off the dead pollen on the afternoon before the day of harvest and re-bagged the tassels to prevent contamination with pollen from neighboring plants. MP viability was tested by *in vitro* germination assays. As shown in Additional file 14, about 90% of pollen grains germinated, and their pollen tubes were long and healthy. After the silks had grown from the husks for approximately 3 days, the upper parts (about 4 cm) were harvested as MS tissues. After removing surrounding glumes and upper silk, whole MO were harvested from the middle portion of the ear. Reproductive tissues (including MP, MO, and MS) used for microarray analysis were similarly collected from other plots at the Shandong Agricultural University experimental station. Samples for each biological replicate were collected from three maize plants and combined. Visible MS, SL, and MO growth conditions were photographed with a Cannon digital camera. MP images were obtained using an Olympus DX51 microscope. All samples were immediately frozen in liquid nitrogen and stored at −80°C for later use.

Total RNA from MS, MP, MO, and SL was extracted according to the CTAB protocol [79] and purified using an RNeasy MinElute Cleanup Kit (Qiagen, Valencia, CA). Purified RNA samples were then quantified by Nanodrop spectrophotometry (Nanodrop Technologies, Wilmington, DE) and agarose gel electrophoresis. RNA integrity was assessed qualitatively using an Agilent 2100 Bioanalyzer (Agilent Technologies, Böbelingen, Germany).

### RNA sequencing library construction and Illumina sequencing

Approximately 5 to 8 μg total RNA was used to construct each RNA-seq library of MS, MP, MO, and SL. Poly(A) RNAs were isolated from total RNA using oligo(dT) magnetic beads (Illumina, San Diego, CA). RNA fragmentation, cDNA synthesis, and PCR amplification were performed according to the Illumina RNA-seq protocol (Cat # RS-100-0801). Sequencing was carried out on an Illumina HiSeq 2000 sequence analyzer at Beijing Genomics Institute (Shenzhen, China). Single-end 49-bp sequence reads were generated. Image data acquired from the sequencing run were base-called and quality-analyzed with Illumina Genome Analysis Pipeline version 1.6. Raw reads with adaptors, containing more than 10% of unknown bases, or with more than 50% of low quality bases (quality value ≤ 5) were excluded from further analysis. To evaluate the sequencing data, we performed a sequence saturation analysis for the data from each of the four tissues. All sequence data are available in the ArrayExpress database [80] under accession number E-MTAB-964.

For reads-to-reference genome/genes alignment, SOAP2 [17] was used, allowing no more than two mismatched bases. Sequence reads for each tissue sample were generated from statistical analysis, and aligned to the AGPv2 maize B73 reference genome [16]. Distributions of reads mapped to genomes and genes from the four tissues are shown in Additional file 2 and Additional file 3. For all mapped genes with unique matched reads, transcript abundance was normalized by using the RPKM (Reads Per kb per Million reads) method [81]. If there was more than one transcript for a gene, the longest one was used to calculate its transcript abundance and coverage.

PCC analyses of log_2_-transformed RPKM values between the four RNA-seq libraries were performed using the R package [82], and log_2_-transformed RPKM values of some genes less than zero were still set to zero. Hierarchical clustering of genes expressed in at least one sample was performed using average linkage cluster analysis (Cluster 3.0), and the results were visualized using Java Treeview 1.0 software (Stanford University School of Medicine, Stanford, CA). A heat map of correlation values was drawn using Scalable Vector Graphics.

### Target preparation, Affymetrix GeneChip hybridization, and statistical analysis of microarray data

In accordance with the Affymetrix GeneChip Expression Analysis Technical Manual [83], 8 μg of total RNA was biotin-labeled for each biological replicate. Target hybridization, washing, staining, and microarray scanning were conducted according to the standard GeneChip® Expression Analysis Technical Manual [83]. Original signal data from microarrays were processed using the GeneChip Operating software (GCOS 1.4). The Affymetrix library RMA (Robust Microarray Analysis) [84] tool was used to normalize the raw data. Microarray data are available in the ArrayExpress database [80] under accession number E-MEXP-3292.

### Real-time PCR analysis

RNA extraction and purification were performed as described above. Genomic DNA contamination was eliminated by RNase-free DNase I treatment (Promega, Madison, WI). Total RNA (4 μg) was reverse-transcribed with oligo (dT) primer using M-MLV reverse transcriptase according to the manufacturer’s instructions (Promega, Madison, WI, USA). All primer sequences are listed in Additional file 7. Quantitative PCR assays in triplicate were performed using SYBR Green Real-time PCR Master Mix (Toyobo, Osaka, Japan) with a Bio-Rad CFX96 Real-Time Detection System. Quantitative variation in the different replicates was calculated using the delta-delta threshold cycle relative quantification method. Amplification of 18S rRNA was used as an internal control for data normalization.

### Comparative analysis of RNA-seq, microarray, and real-time PCR data

To assess the reliability of the RNA-seq results, a comparison between RNA-seq and 18 K Maize Genome array data was carried out. Because the microarray was based on NCBI GenBank (September 29, 2004) and *Z. mays* UniGene Build 42 (July 23, 2004) databases, each probe set was assigned to one EST accession number in GenBank. For the microarray, 17,734 probes represented 13,339 genes in the maize genome [85]. To improve the reliability of the comparison, the corresponding nucleotide sequences were extracted from the Affymetrix GeneChip Maize Genome Array sequence file [85] and blasted against the maize AGPv2 5b filtered gene set database [16]. Using identity > 0.8 and E-value < 1e-20 for the cutoff, the best hit obtained for a given maize transcript was assigned to the gene represented by each probe set. Prior to performing the comparison between RNA-seq and microarray data, genes with more than one predicted isoform were removed from both datasets. RPKM values and RMA-normalized probe set expression values were log_2_-transformed, and SCC values were calculated to account for the discrepancy in scale between the two platforms. In addition, we calculated the correlation coefficient between real-time PCR and RNA-seq results.

### GO analysis

To facilitate comparison of overrepresented GO terms in the four experimental tissues, PAGE [18], a comparative GO analysis tool, was adopted. For genes specifically/preferentially expressed in MS, genes common to maize and rice stigma-specific/preferential datasets, and genes distinct in the MS-specific/preferential dataset, another GO analysis tool, SEA [18], was used to identify overrepresented GO terms.

### Gene annotation, classification, and sub-cellular location prediction

Genes were first annotated using AGPv2 5b.60 [16], and then classified according to MapMan annotation [20] on the longest transcripts of each gene. The sub-cellular location of each gene was predicted using TargetP [86]. To see whether a sub-cellular location was overrepresented, the proteins encoded by the longest transcripts of maize AGPv2 5b filtered datasets [16] were set as a background for the analysis.

### Comparison of stigma-specific/preferential datasets among maize, rice, and Arabidopsis

The specifically or preferentially expressed datasets of maize, rice, and Arabidopsis were compared to identify the similarity and diversity of transcript abundance profiles in their stigmas. The specifically/preferentially expressed genes of rice and Arabidopsis stigmas were acquired directly from published data [12,13], and their annotations were updated by using Rice Genome Annotation Project release 7 [66] and Arabidopsis Information Resource version 10 [41]. Comparisons between maize and rice/Arabidopsis stigma-enriched genes were carried out using two statistical methods. First, protein sequences of maize silk-enriched genes were extracted and blasted against rice and Arabidopsis [41,66] protein databases, with E-values ≤ 1e-10 used to identify their homologous genes in rice and Arabidopsis*.* Using the same criterion, protein sequences of rice and Arabidopsis stigma specifically or preferentially expressed genes were blasted against the maize AGPv2 5b filtered gene set peptide database [16] to find their homologous genes in maize silk. If a homologous gene in one stigma dataset was found in one of the other two stigma datasets, it was considered to be conserved between the two stigma datasets. Secondly, we used function-classification methods to identify conserved and novel gene families among the three species based on MapMan annotation [20].

### Identification of CRPs in the maize silk-specific/preferential dataset

The sequence motif models of each group of CRPs constructed by Silverstein *et al.*[63] were used as queries to blast against the AGPv2 5b filtered gene set peptide database [16]. Using E-value < 1e-10 as the criterion, we obtained a large number of genes with high similarities to the input sequence motif models. We then identified candidate genes belonging to the MS-specific/preferential dataset from these homologs to examine whether they truly encoded CRPs. Genes that met the standards set by Silverstein *et al.*[63] were considered to be novel CRPs in maize silk.

### Comparative analysis of maize transcriptomic data between different inbred lines

We first extracted genes exclusively or preferentially expressed in silk compared with those in post-emergence cob, pollen, and leaf from previously-reported RNA-seq data [15]. Genes expressed in silk but not in the other three tissues were considered to be MS-specific, and genes expressed in maize silk with transcript abundances twice greater than all the other three tissues were designated as MS-preferential. Our MS-specific/preferential dataset was then searched for these specific/preferential genes. Only identical sequences with the same ID numbers were selected.

To compare the MS-specific/preferential dataset with those of tissues absent in our study but present in previously-reported microarray data [14], we first searched for our MS-specific/preferential genes in the microarray data and selected identical genes with the same ID numbers. Using the previously-reported data, the transcript abundance of these genes in silk was then compared with that in roots, whole seedling, internodes, cobs, tassels, anthers, leaves, endosperms, embryos, and pericarp using Significance Analysis of Microarrays software [87]. Genes that were not expressed in other tissues were considered to be specifically expressed in silk. Genes up-regulated in silk with fold change ≥ 2 and q-value ≤ 0.05 were regarded as preferentially expressed.

For comparisons between the MS-specific/preferential dataset and those of tissues absent in our study but analyzed in published RNA-seq data [15], the published data set was searched for our MS-specific/preferential genes, and identical sequences with the same ID numbers were selected. Transcript abundance of these genes in silk was compared with that in leaves, cobs, ovule, tassels, anthers, pollen, seeds, embryo, and endosperm in the published data set. Genes expressed in silk and absent in other tissues were considered to be specifically expressed in silk. Silk-preferential genes were selected using a criterion of transcript abundances in silk twice greater than in all the other tissues.

## Competing interests

The authors declare that they have no competing interests.

## Authors’ contributions

XSZ was responsible for design and coordination in this study. XHX, HC, and JPM performed molecular experiments (material collection, RNA extraction, real-time PCR). XHX and HC carried out the data analysis. XSZ, HC, XHX, and YLS draft this manuscript. XSZ, XQG and FW revised the manuscript. All the authors read and approved the final manuscript.

## Supplementary Material

Additional file 1 **Assessment of the raw sequencing results of MS, MP, MO and SL libraries.** A, Sequencing quality evaluation. Raw reads were classified into four different kinds of reads as adaptor only, N-containing, low quality and clean reads. The first three kinds of reads were removed, and only clean reads were used for further analysis; B, Sequencing saturation analysis. In the beginning of the sequencing, with the number of reads increasing, the number of detected genes is increasing. However, when the number of reads reaches certain value, the growth rate of detected genes flattens, indicating that the number of detected genes tends to saturation; C, Distribution of reads on reference genes. Since reference genes have various lengths, the reads per location on a gene is standardized to a relative position, and the number of reads in each position is counted. If the reads in every position is evenly distributed, the randomness is good. Click here for file

Additional file 2 **Distribution of the experimental tags sequenced in reference maize genome and gene database from the four libraries.** 1a, summary of tag-to-genome mapping data; 1b, summary of tag-to-gene mapping data. Click here for file

Additional file 3 **Distribution of reads on maize inbred line B73 genome.** The distribution of genes and mapped reads on each chromosome were shown in three pictures (green, red and blue). In Gene Number picture (blue), every window (total 500 windows) contains the number of genes on the corresponding nucleotide region (the number in the round bracket). “Coverage” means the ratio of length reads covered in the length of every window. “Log_2_Reads Number” means the binary logarithm of the average sequencing depth of every window. Click here for file

Additional file 4 **Genes expressed in MS, MP, MO and SL.** Locus, Locus identifier in AGPv2 5b filtered gene set transcripts database (Maize Genome Sequence Project, 2011); Uniq_reads_num, the number of reads that uniquely mapped to genes; Coverage, the percentage of a gene covered by reads; RPKM, gene expression; Annotation, the annotations were derived from AGPv2 annotation database directly. Click here for file

Additional file 5 Pearson Correlation Coefficient (PCC) analysis of four studied tissues. Click here for file

Additional file 6 **Cross-comparison analysis of genes expressed in MS, MP, MO and SL results by PAGE.** All genes expressed in at least one of the four tissues were analyzed by PAGE, and the comparison result is displayed in HTML table mode. Accompanied by the color in the block changed from gray to red, gene expression level increased. The FDR value of each term determines the degree of color saturation of the corresponding box. Click here for file

Additional file 7 A list of real-time PCR primers used in this study. Click here for file

Additional file 8 **Stigma specifically/preferentially expressed genes in maize, rice and Arabidopsis.** Rice and Arabidopsis stigma specifically/preferentially expressed genes were derived directly from the previous studies [12,13]. Gene category of maize, rice and Arabidopsis stigma-specific/preferential genes were based on MapMan annotation. Click here for file

Additional file 9 Subcellular localization prediction of MS-specific/preferential genes and the longest transcripts of all filtered genes in maize AGPv2 5b. Click here for file

Additional file 10 Distribution of cysteine-rich proteins in maize, rice and Arabidopsis stigma-specific/preferential datasets. Click here for file

Additional file 11 **Sequence comparison among maize, rice and Arabidopsis stigma-specific/preferential datasets.** A, 471 maize silk specifically/preferentially expressed genes hit 213 rice stigma specific/preferential genes; B, 140 maize silk specifically/preferentially expressed genes hit 37 Arabidopsis stigma-specific/preferential genes; C, 221 rice stigma specifically/preferentially expressed genes hit 179 maize silk-specific/preferential genes; D, 53 Arabidopsis stigma specifically/preferentially expressed genes hit 48 maize silk-specific/preferential expressed genes. Click here for file

Additional file 12 **Distribution of the homologs of maize silk specifically/preferentially expressed genes.** A, Common genes in the three species; B, Maize silk-specific/preferential genes that can only hit rice stigma-specific/preferential dataset; C, Distinct stigma-specific/preferential genes that only expressed in maize silk. Click here for file

Additional file 13 Comparative analysis of maize transcriptomic data between inbred lines Zheng58 and B73. Click here for file

Additional file 14 Mature pollen grains germinate well in liquid medium. Click here for file

## References

[B1] Hiscock SJ Allen AM Diverse cell signalling pathways regulate pollen-stigma interactions: the search for consensus New Phytol 2008 179 2 286 317 10.1111/j.1469-8137.2008.02457.x 19086285

[B2] Sanchez AM Bosch M Bots M Nieuwland J Feron R Mariani C Pistil factors controlling pollination Plant Cell 2004 16 Suppl S98 S106 10.1105/tpc.017806 15010514 PMC2643392

[B3] Swanson R Edlund AF Preuss D Species specificity in pollen-pistil interactions Annu Rev Genet 2004 38 793 818 10.1146/annurev.genet.38.072902.092356 15568994

[B4] Edlund AF Swanson R Preuss D Pollen and stigma structure and function: the role of diversity in pollination Plant Cell 2004 16 Suppl S84 S97 10.1105/tpc.015800 15075396 PMC2643401

[B5] Dresselhaus T Lausser A Márton ML Using maize as a model to study pollen tube growth and guidance, cross-incompatibility and sperm delivery in grasses Ann Bot 2011 108 4 727 737 10.1093/aob/mcr017 21345919 PMC3170146

[B6] Kroh M Gorissen MH Pfahler PL Ultrastructural studies on styles and pollen tubes of Zea mays L. General survey on pollen tube growth in vivo Acta Bot Neerl 1979 28 513 518

[B7] Heslop-Harrison Y Reger BJ Heslop-Harrison J The pollen-stigma interaction in the grasses. 5. Tissue organisation and cytochemistry of the stigma (“silk”) of Zea mays L Acta Bot Neerl 1984 33 81 99

[B8] Wang Z Gerstein M Snyder M RNA-Seq: a revolutionary tool for transcriptomics Nat Rev Genet 2009 10 1 57 63 10.1038/nrg2484 19015660 PMC2949280

[B9] Lord EM Russell SD The mechanisms of pollination and fertilization in plants Annu Rev Cell Dev Biol 2002 18 81 105 10.1146/annurev.cellbio.18.012502.083438 12142268

[B10] Takeuchi H Higashiyama T Attraction of tip-growing pollen tubes by the female gametophyte Curr Opin Plant Biol 2011 14 5 614 621 10.1016/j.pbi.2011.07.010 21855396

[B11] Swanson R Clark T Preuss D Expression profiling of Arabidopsis stigma tissue identifies stigma-specific genes Sex Plant Reprod 2005 18 4 163 171 10.1007/s00497-005-0009-x

[B12] Tung CW Dwyer KG Nasrallah ME Nasrallah JB Genome-wide identification of genes expressed in Arabidopsis pistils specifically along the path of pollen tube growth Plant Physiol 2005 138 2 977 989 10.1104/pp.105.060558 15894741 PMC1150412

[B13] Li M Xu W Yang W Kong Z Xue Y Genome-wide gene expression profiling reveals conserved and novel molecular functions of the stigma in rice Plant Physiol 2007 144 4 1797 1812 10.1104/pp.107.101600 17556504 PMC1949881

[B14] Sekhon RS Lin H Childs KL Hansey CN Buell CR de Leon N Kaeppler SM Genome-wide atlas of transcription during maize development Plant J 2011 66 4 553 563 10.1111/j.1365-313X.2011.04527.x 21299659

[B15] Davidson RM Hansey CN Gowda M Childs KL Lin H Vaillancourt B Sekhon RS de Leon N Kaeppler SM Jiang N Buell CR Utility of RNA Sequencing for Analysis of Maize Reproductive Transcriptomes Plant Genome 2011 4 191 203 10.3835/plantgenome2011.05.0015

[B16] Maize genome sequencing project http://www.maizesequence.org/index.html

[B17] Li R Yu C Li Y Lam TW Yiu SM Kristiansen K Wang J SOAP2: an improved ultrafast tool for short read alignment Bioinformatics 2009 25 15 1966 1967 10.1093/bioinformatics/btp336 19497933

[B18] Du Z Zhou X Ling Y Zhang Z Su Z agriGO: a GO analysis toolkit for the agricultural community Nucleic Acids Res 2010 38 2 W64 70 20435677 10.1093/nar/gkq310 PMC2896167

[B19] Audic S Claverie JM The significance of digital gene expression profiles Genome Res 1997 7 10 986 995 9331369 10.1101/gr.7.10.986

[B20] Thimm O Bläsing O Gibon Y Nagel A Meyer S Krüger P Selbig J Müller LA Rhee SY Stitt M MAPMAN: a user-driven tool to display genomics data sets onto diagrams of metabolic pathways and other biological processes Plant J 2004 37 6 914 939 10.1111/j.1365-313X.2004.02016.x 14996223

[B21] Hennig L Gruissem W Grossniklaus U Köhler C Transcriptional programs of early reproductive stages in Arabidopsis Plant Physiol 2004 135 3 1765 1775 10.1104/pp.104.043182 15247381 PMC519088

[B22] Chandran D Tai YC Hather G Dewdney J Denoux C Burgess DG Ausubel FM Speed TP Wildermuth MC Temporal global expression data reveal known and novel salicylate-impacted processes and regulators mediating powdery mildew growth and reproduction on Arabidopsis Plant Physiol 2009 149 3 1435 1451 10.1104/pp.108.132985 19176722 PMC2649394

[B23] Dubos C Stracke R Grotewold E Weisshaar B Martin C Lepiniec L MYB transcription factors in Arabidopsis Trends Plant Sci 2010 15 10 573 581 10.1016/j.tplants.2010.06.005 20674465

[B24] Reňák D Dupl’áková N Honys D Wide-scale screening of T-DNA lines for transcription factor genes affecting male gametophyte development in Arabidopsis Sex Plant Reprod 2011 25 1 39 60 22101548 10.1007/s00497-011-0178-8

[B25] Charlesworth D Vekemans X Castric V Glémin S Plant self-incompatibility systems: a molecular evolutionary perspective New Phytol 2005 168 1 61 69 10.1111/j.1469-8137.2005.01443.x 16159321

[B26] Stone SL Anderson EM Mullen RT Goring DR ARC1 is an E3 ubiquitin ligase and promotes the ubiquitination of proteins during the rejection of self-incompatible Brassica pollen Plant Cell 2003 15 4 885 898 10.1105/tpc.009845 12671085 PMC152337

[B27] Ushijima K Sassa H Dandekar AM Gradziel TM Tao R Hirano H Structural and transcriptional analysis of the self-incompatibility locus of almond: identification of a pollen-expressed F-box gene with haplotype-specific polymorphism Plant Cell 2003 15 3 771 781 10.1105/tpc.009290 12615948 PMC150029

[B28] Sakai N Sawada MT Sawada H Non-traditional roles of ubiquitin-proteasome system in fertilization and gametogenesis Int J Biochem Cell Biol 2004 36 5 776 784 10.1016/S1357-2725(03)00263-2 15006630

[B29] Sutovsky P Moreno R Ramalho-Santos J Dominko T Thompson WE Schatten G A putative, ubiquitin-dependent mechanism for the recognition and elimination of defective spermatozoa in the mammalian epididymis J Cell Sci 2001 114 Pt 9 1665 1675 11309198 10.1242/jcs.114.9.1665

[B30] Shiu SH Bleecker AB Expansion of the receptor-like kinase/Pelle gene family and receptor-like proteins in Arabidopsis Plant Physiol 2003 132 2 530 543 10.1104/pp.103.021964 12805585 PMC166995

[B31] Chen K Fan B Du L Chen Z Activation of hypersensitive cell death by pathogen-induced receptor-like protein kinases from Arabidopsis Plant Mol Biol 2004 56 2 271 283 10.1007/s11103-004-3381-2 15604743

[B32] Acharya BR Raina S Maqbool SB Jagadeeswaran G Mosher SL Appel HM Schultz JC Klessig DF Raina R Overexpression of CRK13, an Arabidopsis cysteine-rich receptor-like kinase, results in enhanced resistance to Pseudomonas syringae Plant J 2007 50 3 488 499 10.1111/j.1365-313X.2007.03064.x 17419849

[B33] Thomas CL Bayer EM Ritzenthaler C Fernandez-Calvino L Maule AJ Specific targeting of a plasmodesmal protein affecting cell-to-cell communication PLoS Biol 2008 6 1 e7 10.1371/journal.pbio.0060007 18215111 PMC2211546

[B34] Yang T Chaudhuri S Yang L Chen Y Poovaiah BW Calcium/calmodulin up-regulates a cytoplasmic receptor-like kinase in plants J Biol Chem 2004 279 41 42552 42559 10.1074/jbc.M402830200 15292241

[B35] Hepler PK Kunkel JG Rounds CM Winship LJ Calcium entry into pollen tubes Trends Plant Sci 2011 17 1 32 38 22104406 10.1016/j.tplants.2011.10.007

[B36] Zhang Y McCormick S The regulation of vesicle trafficking by small GTPases and phospholipids during pollen tube growth Sex Plant Reprod 2010 23 2 87 93 10.1007/s00497-009-0118-z 20490965

[B37] Tang W Kelley D Ezcurra I Cotter R McCormick S LeSTIG1, an extracellular binding partner for the pollen receptor kinases LePRK1 and LePRK2, promotes pollen tube growth in vitro Plant J 2004 39 3 343 353 10.1111/j.1365-313X.2004.02139.x 15255864

[B38] Zou Y Aggarwal M Zheng WG Wu HM Cheung AY Receptor-like kinases as surface regulators for RAC/ROP-mediated pollen tube growth and interaction with the pistil AoB Plants 2011 plr017 10.1093/aobpla/plr017 PMC3158858 22476487

[B39] Rea AC Nasrallah JB Self-incompatibility systems: barriers to self-fertilization in flowering plants Int J Dev Biol 2008 52 5–6 627 636 18649276 10.1387/ijdb.072537ar

[B40] Gao X-Q Zhu D Zhang X Stigma factors regulating self-compatible pollination Front Biol 2010 5 2 156 163 10.1007/s11515-010-0024-7

[B41] The Arabidopsis Information Resource http://www.arabidopsis.org/

[B42] Boisson-Dernier A Frietsch S Kim TH Dizon MB Schroeder JI The peroxin loss-of-function mutation abstinence by mutual consent disrupts male–female gametophyte recognition Curr Biol 2008 18 1 63 68 10.1016/j.cub.2007.11.067 18160292 PMC2278027

[B43] Wolters-Arts M Lush WM Mariani C Lipids are required for directional pollen-tube growth Nature 1998 392 6678 818 821 10.1038/33929 9572141

[B44] Wolters-Arts M Van Der Weerd L Van Aelst AC Van Der Weerd J Van As H Mariani C Water-conducting properties of lipids during pollen hydration Plant Cell Environ 2002 25 4 513 519 10.1046/j.1365-3040.2002.00827.x

[B45] Hülskamp M Kopczak SD Horejsi TF Kihl BK Pruitt RE Identification of genes required for pollen-stigma recognition in Arabidopsis thaliana Plant J 1995 8 5 703 714 10.1046/j.1365-313X.1995.08050703.x 8528281

[B46] Lessire R Cahoon E Chapman K Dyer J Eastmond P Heinz E Highlights of recent progress in plant lipid research Plant Physiol Biochem 2009 47 6 443 447 10.1016/j.plaphy.2009.02.010 19328004

[B47] Preuss D Lemieux B Yen G Davis RW A conditional sterile mutation eliminates surface components from Arabidopsis pollen and disrupts cell signaling during fertilization Genes Dev 1993 7 6 974 985 10.1101/gad.7.6.974 8504936

[B48] Pruitt RE Vielle-Calzada JP Ploense SE Grossniklaus U Lolle SJ FIDDLEHEAD, a gene required to suppress epidermal cell interactions in Arabidopsis, encodes a putative lipid biosynthetic enzyme Proc Natl Acad Sci U S A 2000 97 3 1311 1316 10.1073/pnas.97.3.1311 10655527 PMC15605

[B49] Lolle SJ Cheung AY Promiscuous germination and growth of wildtype pollen from Arabidopsis and related species on the shoot of the Arabidopsis mutant, fiddlehead Dev Biol 1993 155 1 250 258 10.1006/dbio.1993.1022 8416837

[B50] Meijer HJ Munnik T Phospholipid-based signaling in plants Annu Rev Plant Biol 2003 54 265 306 10.1146/annurev.arplant.54.031902.134748 14502992

[B51] Park SY Lord EM Expression studies of SCA in lily and confirmation of its role in pollen tube adhesion Plant Mol Biol 2003 51 2 183 189 10.1023/A:1021139502947 12602877

[B52] Kim ST Zhang K Dong J Lord EM Exogenous free ubiquitin enhances lily pollen tube adhesion to an in vitro stylar matrix and may facilitate endocytosis of SCA Plant Physiol 2006 142 4 1397 1411 10.1104/pp.106.086801 16998086 PMC1676050

[B53] Chae K Zhang K Zhang L Morikis D Kim ST Mollet JC de la Rosa N Tan K Lord EM Two SCA (stigma/style cysteine-rich adhesin) isoforms show structural differences that correlate with their levels of in vitro pollen tube adhesion activity J Biol Chem 2007 282 46 33845 33858 10.1074/jbc.M703997200 17878166

[B54] Updegraff EP Zhao F Preuss D The extracellular lipase EXL4 is required for efficient hydration of Arabidopsis pollen Sex Plant Reprod 2009 22 3 197 204 10.1007/s00497-009-0104-5 20033440

[B55] Kumar A McClure B Pollen-pistil interactions and the endomembrane system J Exp Bot 2010 61 7 2001 2013 10.1093/jxb/erq065 20363870

[B56] Michard E Lima PT Borges F Silva AC Portes MT Carvalho JE Gilliham M Liu LH Obermeyer G Feijó JA Glutamate receptor-like genes form Ca2+ channels in pollen tubes and are regulated by pistil d-Serine Science 2011 332 6028 434 437 10.1126/science.1201101 21415319

[B57] Komarova NY Thor K Gubler A Meier S Dietrich D Weichert A Suter Grotemeyer M Tegeder M Rentsch D AtPTR1 and AtPTR5 transport dipeptides in planta Plant Physiol 2008 148 2 856 869 10.1104/pp.108.123844 18753286 PMC2556804

[B58] Tegeder M Rentsch D Uptake and partitioning of amino acids and peptides Mol Plant 2010 3 6 997 1011 10.1093/mp/ssq047 21081651

[B59] Bednarska E Calcium uptake from the stigma by germinating pollen in Primula officinalis L. and Ruscus aculeatus L Sex Plant Reprod 1991 4 1 36 38

[B60] Zienkiewicz K Rejón JD Suárez C Castro AJ de Dios Alché J Rodríguez Garciá MI Whole-Organ analysis of calcium behavior in the developing pistil of olive (Olea europaea L.) as a tool for the determination of key events in sexual plant reproduction BMC Plant Biol 2011 11 150 10.1186/1471-2229-11-150 22050767 PMC3228850

[B61] Holdaway-Clarke TL Hepler PK Control of pollen tube growth: role of ion gradients and fluxes New Phytol 2003 159 3 539 563 10.1046/j.1469-8137.2003.00847.x 33873604

[B62] Higashiyama T Peptide signaling in pollen-pistil interactions Plant Cell Physiol 2010 51 2 177 189 10.1093/pcp/pcq008 20081210

[B63] Silverstein KA Moskal WA Wu HC Underwood BA Graham MA Town CD VandenBosch KA Small cysteine-rich peptides resembling antimicrobial peptides have been under-predicted in plants Plant J 2007 51 2 262 280 10.1111/j.1365-313X.2007.03136.x 17565583

[B64] Mayfield JA Preuss D Rapid initiation of Arabidopsis pollination requires the oleosin-domain protein GRP17 Nat Cell Biol 2000 2 2 128 130 10.1038/35000084 10655594

[B65] Ryan C Protease inhibitors in plants: Genes for improving defenses against insects and pathogens Annu Rev Phytopathol 1990 28 425 449 10.1146/annurev.py.28.090190.002233

[B66] MSU Rice Genome Annotation Project http://rice.plantbiology.msu.edu/

[B67] Luu DT Marty-Mazars D Trick M Dumas C Heizmann P Pollen-stigma adhesion in Brassica spp involves SLG and SLR1 glycoproteins Plant Cell 1999 11 2 251 262 9927642 10.1105/tpc.11.2.251 PMC144154

[B68] Dixit R Rizzo C Nasrallah M Nasrallah J The Brassica MIP-MOD gene encodes a functional water channel that is expressed in the stigma epidermis Plant Mol Biol 2001 45 1 51 62 10.1023/A:1006428007826 11247606

[B69] Bosch M Cheung AY Hepler PK Pectin methylesterase, a regulator of pollen tube growth Plant Physiol 2005 138 3 1334 1346 10.1104/pp.105.059865 15951488 PMC1176407

[B70] Jiang L Yang SL Xie LF Puah CS Zhang XQ Yang WC Sundaresan V Ye D VANGUARD1 encodes a pectin methylesterase that enhances pollen tube growth in the Arabidopsis style and transmitting tract Plant Cell 2005 17 2 584 596 10.1105/tpc.104.027631 15659637 PMC548828

[B71] Kapu NU Cosgrove DJ Changes in growth and cell wall extensibility of maize silks following pollination J Exp Bot 2010 61 14 4097 4107 10.1093/jxb/erq225 20656797 PMC2935878

[B72] Lai J Li R Xu X Jin W Xu M Zhao H Xiang Z Song W Ying K Zhang M Genome-wide patterns of genetic variation among elite maize inbred lines Nat Genet 2010 42 11 1027 1030 10.1038/ng.684 20972441

[B73] Schiefelbein J Galway M Masucci J Ford S Pollen tube and root-hair tip growth is disrupted in a mutant of Arabidopsis thaliana Plant Physiol 1993 103 3 979 985 10.1104/pp.103.3.979 8022944 PMC159072

[B74] Fiebig A Mayfield JA Miley NL Chau S Fischer RL Preuss D Alterations in CER6, a gene identical to CUT1, differentially affect long-chain lipid content on the surface of pollen and stems Plant Cell 2000 12 10 2001 2008 11041893 10.1105/tpc.12.10.2001 PMC149136

[B75] Majewska-Sawka A Nothnagel EA The multiple roles of arabinogalactan proteins in plant development Plant Physiol 2000 122 1 3 10 10.1104/pp.122.1.3 10631243 PMC1539237

[B76] Procissi A Guyon A Pierson ES Giritch A Knuiman B Grandjean O Tonelli C Derksen J Pelletier G Bonhomme S KINKY POLLEN encodes a SABRE-like protein required for tip growth in Arabidopsis and conserved among eukaryotes Plant J 2003 36 6 894 904 10.1046/j.1365-313X.2003.01933.x 14675453

[B77] Cole RA Synek L Zarsky V Fowler JE SEC8, a subunit of the putative Arabidopsis exocyst complex, facilitates pollen germination and competitive pollen tube growth Plant Physiol 2005 138 4 2005 2018 10.1104/pp.105.062273 16040664 PMC1183391

[B78] Samuel MA Chong YT Haasen KE Aldea-Brydges MG Stone SL Goring DR Cellular pathways regulating responses to compatible and self-incompatible pollen in Brassica and Arabidopsis stigmas intersect at Exo70A1, a putative component of the exocyst complex Plant Cell 2009 21 9 2655 2671 10.1105/tpc.109.069740 19789280 PMC2768929

[B79] Gambino G Perrone I Gribaudo I A Rapid and effective method for RNA extraction from different tissues of grapevine and other woody plants Phytochem Anal 2008 19 6 520 525 10.1002/pca.1078 18618437

[B80] ArrayExpress http://www.ebi.ac.uk/arrayexpress/

[B81] Mortazavi A Williams BA McCue K Schaeffer L Wold B Mapping and quantifying mammalian transcriptomes by RNA-Seq Nat Methods 2008 5 7 621 628 10.1038/nmeth.1226 18516045 PMC13303166

[B82] The R Project for Statistical Computing http://www.r-project.org/

[B83] Expression Analysis Technical Manual, with Specific Protocols for Use with the Hybridization, Wash, and Stain Kit http://media.affymetrix.com/support/downloads/manuals/expression_analysis_manual.pdf

[B84] Irizarry RA Hobbs B Collin F Beazer-Barclay YD Antonellis KJ Scherf U Speed TP Exploration, normalization, and summaries of high density oligonucleotide array probe level data Biostatistics 2003 4 2 249 264 10.1093/biostatistics/4.2.249 12925520

[B85] GeneChip® Maize Genome Array http://www.affymetrix.com/estore/browse/products.jsp?productId=131468#1_1

[B86] TargetP fd http://www.cbs.dtu.dk/services/TargetP-1.0

[B87] Tusher VG Tibshirani R Chu G Significance analysis of microarrays applied to the ionizing radiation response Proc Natl Acad Sci U S A 2001 98 9 5116 5121 10.1073/pnas.091062498 11309499 PMC33173

