# Supplementary figures and images for: Identification of genes specifically or preferentially expressed in maize silk reveals similarity and diversity in transcript abundance of different dry stigmas
Source: BMC Genomics. 2012 Jul 2;13:294. doi: 10.1186/1471-2164-13-294 (PMC3416702; doi:10.1186/1471-2164-13-294)

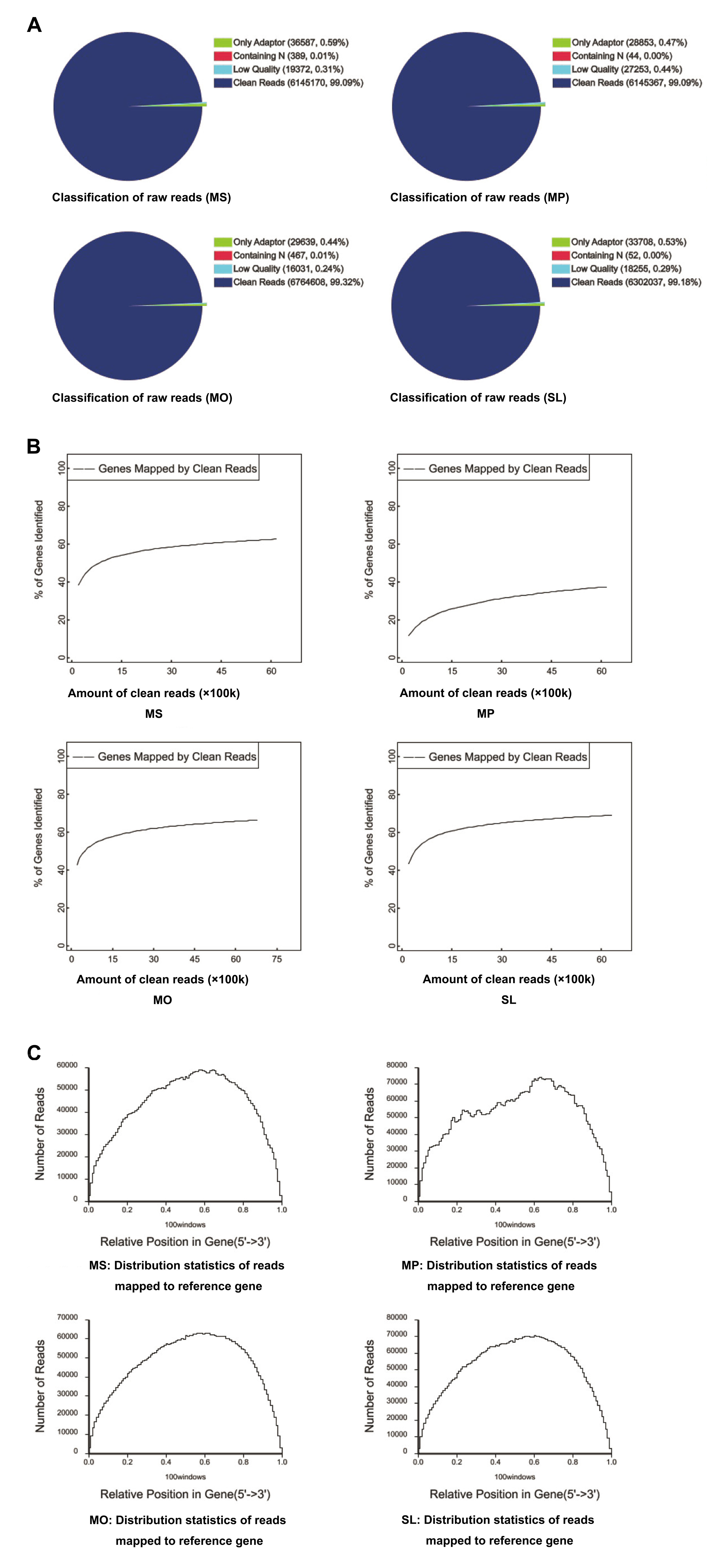

Supplement: Additional file 1 — Assessment of the raw sequencing results of MS, MP, MO and SL libraries. A, Sequencing quality evaluation. Raw reads were classified into four different kinds of reads as adaptor only, N-containing, low quality and clean reads. The first three kinds of reads were removed, and only clean reads were used for further analysis; B, Sequencing saturation analysis. In the beginning of the sequencing, with the number of reads increasing, the number of detected genes is increasing. However, when the number of reads reaches certain value, the growth rate of detected genes flattens, indicating that the number of detected genes tends to saturation; C, Distribution of reads on reference genes. Since reference genes have various lengths, the reads per location on a gene is standardized to a relative position, and the number of reads in each position is counted. If the reads in every position is evenly distributed, the randomness is good. [file 1471-2164-13-294-S1.jpeg]

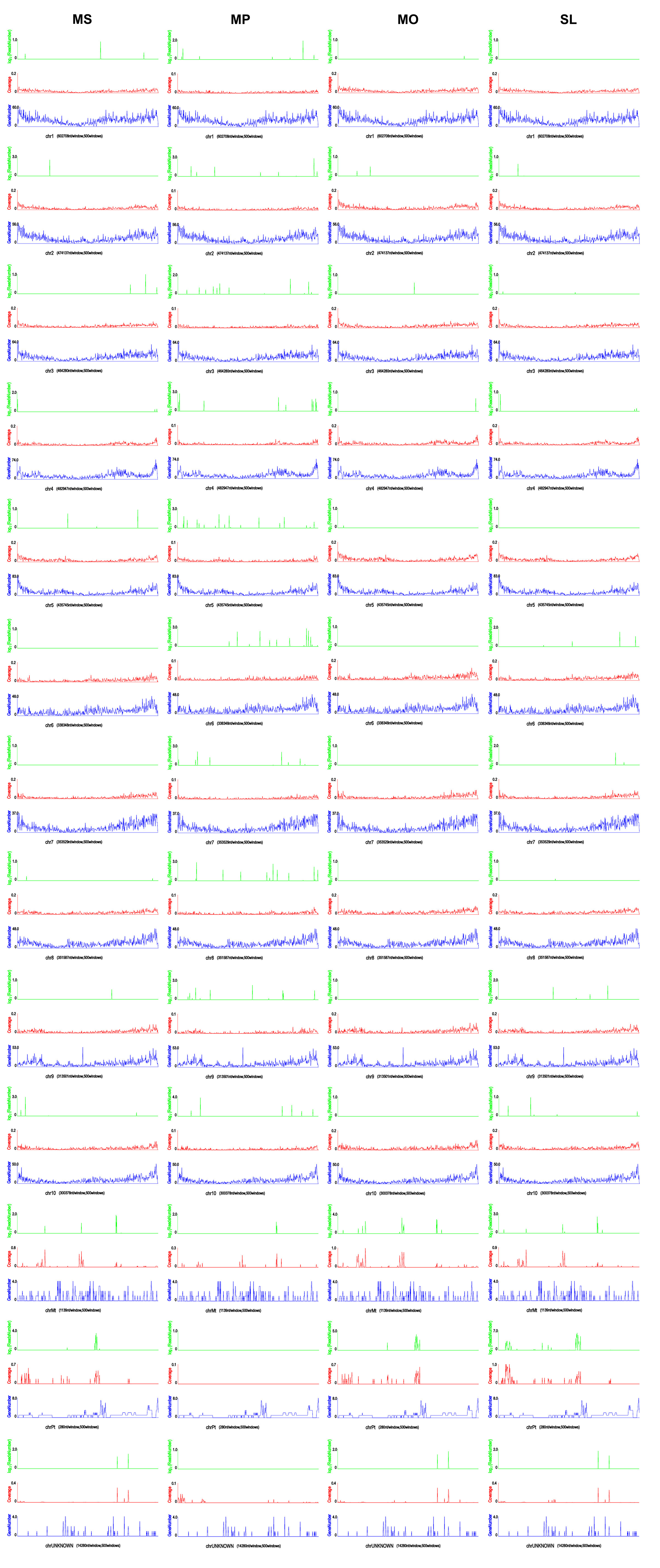

Supplement: Additional file 3 — Distribution of reads on maize inbred line B73 genome. The distribution of genes and mapped reads on each chromosome were shown in three pictures (green, red and blue). In Gene Number picture (blue), every window (total 500 windows) contains the number of genes on the corresponding nucleotide region (the number in the round bracket). “Coverage” means the ratio of length reads covered in the length of every window. “Log2Reads Number” means the binary logarithm of the average sequencing depth of every window. [file 1471-2164-13-294-S3.jpeg]

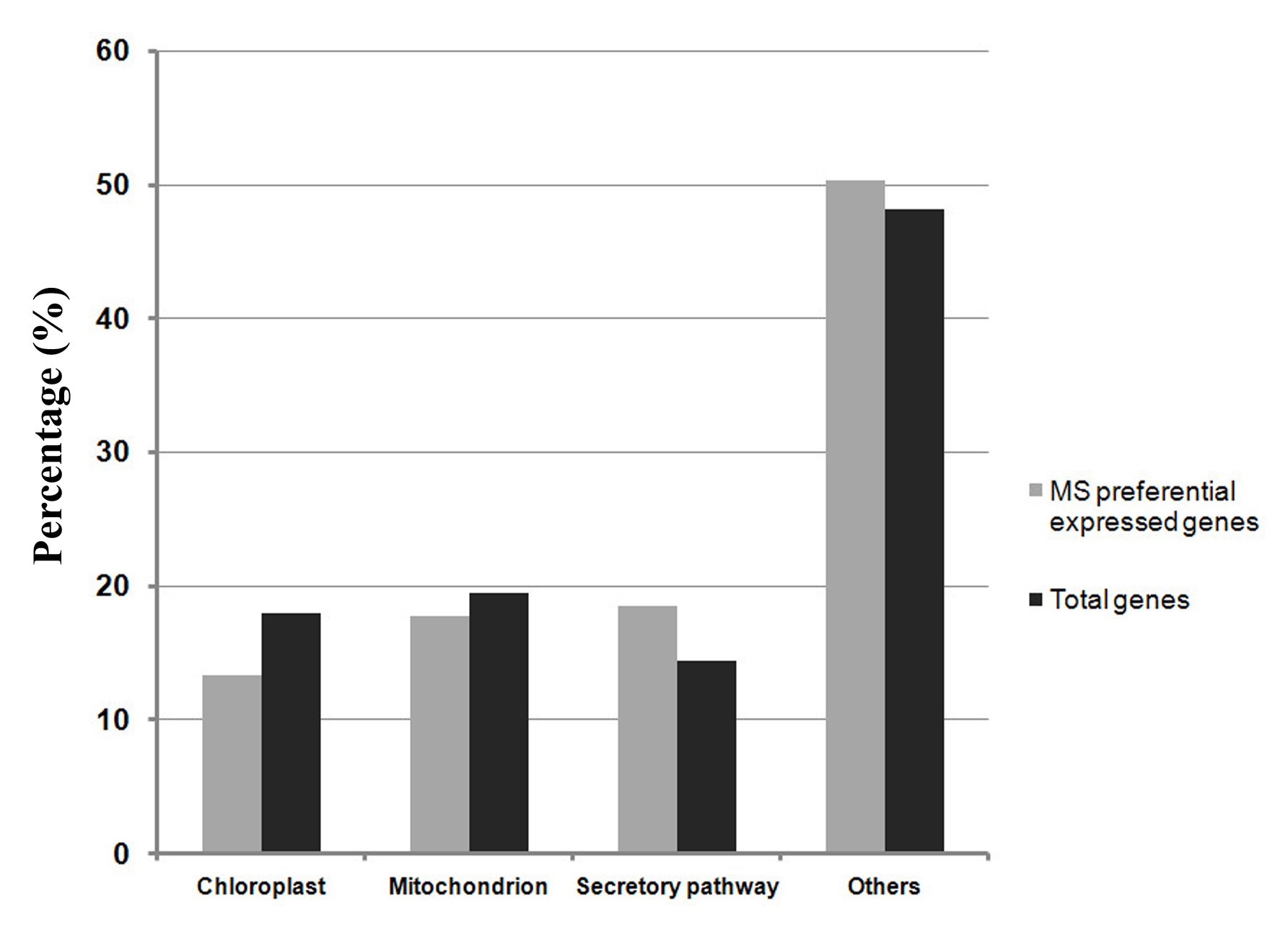

Supplement: Additional file 9 — Subcellular localization prediction of MS-specific/preferential genes and the longest transcripts of all filtered genes in maize AGPv2 5b. [file 1471-2164-13-294-S9.jpeg]

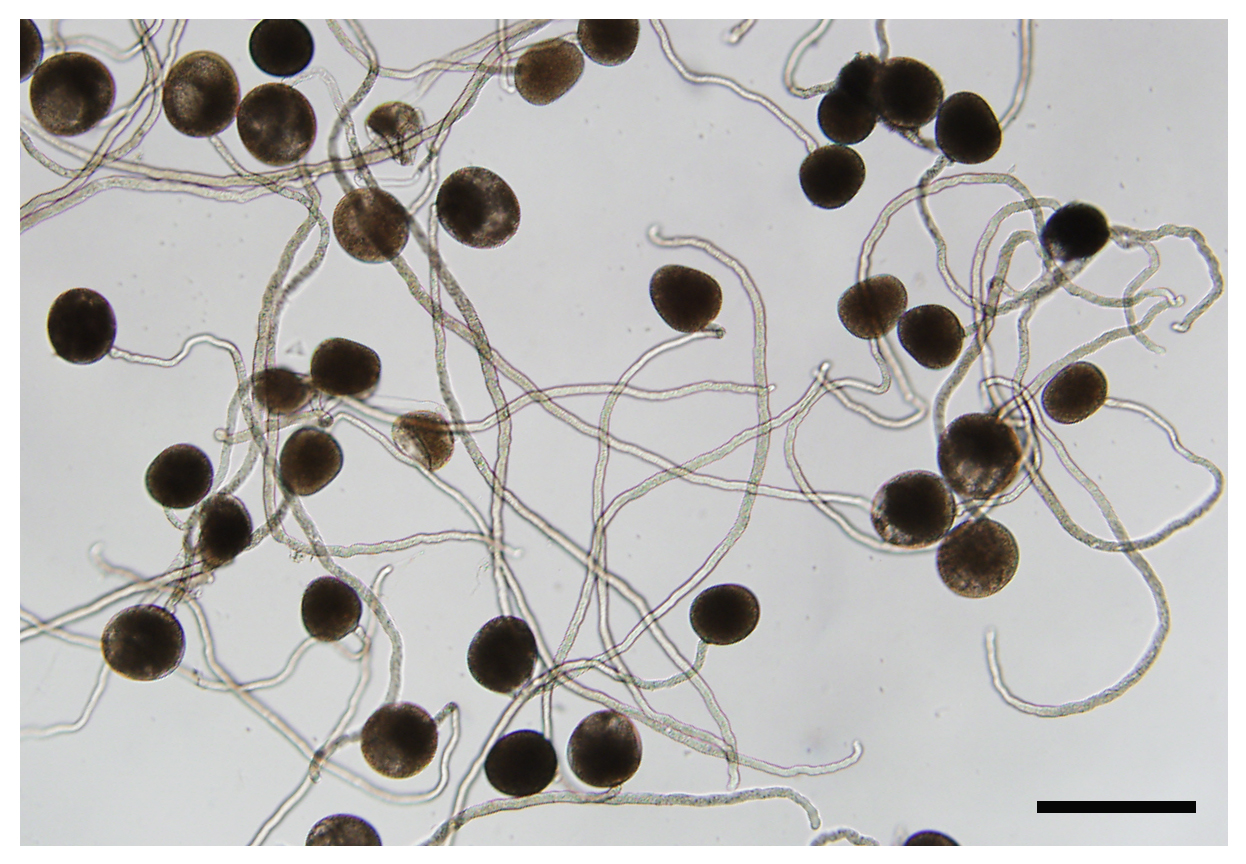

Supplement: Additional file 13 — Comparative analysis of maize transcriptomic data between inbred lines Zheng58 and B73. [file 1471-2164-13-294-S13.jpeg]
