# Supplementary material for: Identification of genes specifically or preferentially expressed in maize silk reveals similarity and diversity in transcript abundance of different dry stigmas
Source: BMC Genomics. 2012 Jul 2;13:294. doi: 10.1186/1471-2164-13-294 (PMC3416702; doi:10.1186/1471-2164-13-294)
Supplement: Additional file 2 — Distribution of the experimental tags sequenced in reference maize genome and gene database from the four libraries. 1a, summary of tag-to-genome mapping data; 1b, summary of tag-to-gene mapping data. [file 1471-2164-13-294-S2.doc]

**Table 1. Distribution of the experimental tags sequenced in reference maize genome and gene database from the four experimental tissue libraries. 1a，summary of tag-to-genome mapping data; 1b, summary of tag-to-gene mapping data.**

**Table 1a**

|  | MS | | MP | | MO | | SL | |
| --- | --- | --- | --- | --- | --- | --- | --- | --- |
|  | reads number | percentage | reads number | percentage | reads number | percentage | reads number | percentage |
| Total Reads | 6145170 | 100.00% | 6145367 | 100.00% | 6764608 | 100.00% | 6302037 | 100.00% |
| Total BasePairs | 301113330 | 100.00% | 301122983 | 100.00% | 331465792 | 100.00% | 308799813 | 100.00% |
| Total Mapped Reads | 4249353 | 69.15% | 5289705 | 86.08% | 5408379 | 79.95% | 4978668 | 79.00% |
| perfect match | 3090125 | 50.29% | 4015216 | 65.34% | 4029406 | 59.57% | 3697755 | 58.68% |
| <=3bp mismatch | 1159228 | 18.86% | 1274489 | 20.74% | 1378973 | 20.39% | 1280913 | 20.33% |
| unique match | 3666501 | 59.66% | 4178820 | 68.00% | 4609075 | 68.14% | 4171051 | 66.19% |
| multi-position match | 582852 | 9.48% | 1110885 | 18.08% | 799304 | 11.82% | 807617 | 12.82% |
| Total Unmapped Reads | 1895817 | 30.85% | 855662 | 13.92% | 1356229 | 20.05% | 1323369 | 21.00% |

**Table 1b**

|  | MS | | MP | | MO | | SL | |
| --- | --- | --- | --- | --- | --- | --- | --- | --- |
|  | reads number | percentage | reads number | percentage | reads number | percentage | reads number | percentage |
| Total Reads | 6145170 | 100.00% | 6145367 | 100.00% | 6764608 | 100.00% | 6302037 | 100.00% |
| Total BasePairs | 301113330 | 100.00% | 301122983 | 100.00% | 331465792 | 100.00% | 308799813 | 100.00% |
| Total Mapped Reads | 4250779 | 69.17% | 5154569 | 83.88% | 5301977 | 78.38% | 5110889 | 81.10% |
| perfect match | 3214764 | 52.31% | 4009491 | 65.24% | 4101657 | 60.63% | 3932496 | 62.40% |
| <=3bp mismatch | 1036015 | 16.86% | 1145078 | 18.63% | 1200320 | 17.74% | 1178393 | 18.70% |
| unique match | 3868626 | 62.95% | 4088802 | 66.53% | 4824179 | 71.31% | 4545796 | 72.13% |
| multi-position match | 382153 | 6.22% | 1065767 | 17.34% | 477780 | 7.06% | 565093 | 8.97% |
| Total Unmapped Reads | 1894391 | 30.83% | 990798 | 16.12% | 1462631 | 21.62% | 1191148 | 18.90% |
