# Supplementary material for: Identification of genes specifically or preferentially expressed in maize silk reveals similarity and diversity in transcript abundance of different dry stigmas
Source: BMC Genomics. 2012 Jul 2;13:294. doi: 10.1186/1471-2164-13-294 (PMC3416702; doi:10.1186/1471-2164-13-294)
Supplement: Additional file 6 — Cross-comparison analysis of genes expressed in MS, MP, MO and SL results by PAGE. All genes expressed in at least one of the four tissues were analyzed by PAGE, and the comparison result is displayed in HTML table mode. Accompanied by the color in the block changed from gray to red, gene expression level increased. The FDR value of each term determines the degree of color saturation of the corresponding box. [file 1471-2164-13-294-S6.pdf]

GO Analysis Toolkit and Database for Agricultural Community

Select terms to [Draw graphical results ☐] [Create bar chart ☐] [Suppress term number ☐]
[Use NO-adjusted Pvalue ☐]

GO Information					CM				☐ MS			☐ MP			☐ MO			☐ SL		
No	☐ GO Term	Onto	Number	Description	1	2	3	4	Z-score	Mean	FDRnbsp;	Z-score	Mean	FDRnbsp;	Z-score	Mean	FDRnbsp;	Z-score	Mean	FDRnbsp;
1	☐ GO:0006032	P	16	chitin catabolic process					15	4.6e+02	0	-0.44	0.58	0.93	-1	12	0.54	-0.56	15	0.81
2	☐ GO:0006030	P	16	chitin metabolic process					15	4.6e+02	0	-0.44	0.58	0.93	-1	12	0.54	-0.56	15	0.81
3	☐ GO:0006026	P	16	aminoglycan catabolic process					15	4.6e+02	0	-0.44	0.58	0.93	-1	12	0.54	-0.56	15	0.81
4	☐ GO:0006022	P	18	aminoglycan metabolic process					14	4.1e+02	0	-0.47	0.52	0.93	-1.1	12	0.48	-0.61	15	0.8
5	☐ GO:0000272	P	28	polysaccharide catabolic process					11	2.6e+02	0	2.3	1.4e+02	0.27	-1.1	14	0.51	-0.65	17	0.79
6	☐ GO:0010876	P	49	lipid localization					7.9	1.5e+02	3e-13	0.3	40	0.93	0.054	23	0.99	0.79	33	0.75
7	☐ GO:0006869	P	49	lipid transport					7.9	1.5e+02	3e-13	0.3	40	0.93	0.054	23	0.99	0.79	33	0.75
8	☐ GO:0009057	P	315	macromolecule catabolic process					5.9	63	3.5e-07	-0.34	24	0.93	6.7	38	3.7e-10	6.5	50	7.9e-10
9	☐ GO:0016052	P	159	carbohydrate catabolic process					5.8	78	3.9e-07	0.58	41	0.93	3.8	35	0.00083	7.2	64	6.7e-12
10	☐ GO:0016998	P	30	cell wall macromolecule catabolic process					5.7	1.4e+02	6.4e-07	-0.47	6.8	0.93	-1.3	13	0.41	-1.3	9.1	0.45
11	☐ GO:0009056	P	423	catabolic process					5.5	55	1.9e-06	-0.75	20	0.93	5.3	33	1.1e-06	6.6	46	4.9e-10
12	☐ GO:0044036	P	32	cell wall macromolecule metabolic process					5.5	1.4e+02	2.2e-06	-0.5	6.4	0.93	-1.4	13	0.36	-1.3	9.4	0.44
13	☐ GO:0009074	P	10	aromatic amino acid family catabolic process					4.2	1.8e+02	0.0011	-0.35	0.14	0.93	-0.17	21	0.94	1.6	60	0.28
14	☐ GO:0006559	P	10	L-phenylalanine catabolic process					4.2	1.8e+02	0.0011	-0.35	0.14	0.93	-0.17	21	0.94	1.6	60	0.28
15	☐ GO:0005976	P	164	polysaccharide metabolic process					4.2	62	0.0011	0.56	41	0.93	2.7	31	0.027	0.94	30	0.65
16	☐ GO:0006633	P	77	fatty acid biosynthetic process					3.8	75	0.0049	-0.54	13	0.93	-0.21	22	0.93	0.76	31	0.75
17	☐ GO:0009308	P	389	amine metabolic process					3.8	47	0.0049	-0.44	23	0.93	3.6	30	0.0016	5	42	3e-06
18	☐ GO:0006631	P	95	fatty acid metabolic process					3.8	70	0.0049	-0.67	11	0.93	-0.052	23	0.99	0.81	31	0.73
19	☐ GO:0033036	P	427	macromolecule localization					3.8	45	0.0057	-0.68	21	0.93	4.1	31	0.00021	1.4	30	0.36
20	☐ GO:0006414	P	44	translational elongation					3.8	89	0.0059	-0.38	14	0.93	13	1e+02	0	14	1.7e+02	0
21	☐ GO:0032787	P	121	monocarboxylic acid metabolic process					3.5	61	0.014	-0.79	11	0.93	-0.099	22	0.97	1.4	34	0.38
22	☐ GO:0051258	P	38	protein polymerization					3.5	89	0.015	3.9	1.9e+02	0.0038	13	1.1e+02	0	6.1	92	8.4e-09
23	☐ GO:0006558	P	16	L-phenylalanine metabolic process					3.2	1.2e+02	0.044	-0.45	0.3	0.93	-0.47	18	0.81	0.99	42	0.62
24	☐ GO:0004568	F	16	chitinase activity					15	4.6e+02	0	-0.44	0.58	0.93	-1	12	0.64	-0.56	15	0.8
25	☐ GO:0008289	F	88	lipid binding					6.2	1e+02	1.3e-07	0.53	44	0.93	0.63	26	0.81	0.71	30	0.77
26	☐ GO:0005199	F	82	structural constituent of cell wall					5.9	1e+02	5e-07	1.1	60	0.93	4.8	45	3.2e-05	0.44	28	0.84
27	☐ GO:0042923	F	16	neuropeptide binding					4.8	1.6e+02	0.00016	-0.44	0.92	0.93	1.2	35	0.54	0.32	30	0.88
28	☐ GO:0008188	F	16	neuropeptide receptor activity					4.8	1.6e+02	0.00016	-0.44	0.92	0.93	1.2	35	0.54	0.32	30	0.88
29	☐ GO:0042165	F	19	neurotransmitter binding					4.5	1.4e+02	0.00059	-0.47	1.1	0.93	1.3	35	0.54	0.3	30	0.89
30	☐ GO:0030594	F	19	neurotransmitter receptor activity					4.5	1.4e+02	0.00059	-0.47	1.1	0.93	1.3	35	0.54	0.3	30	0.89
31	☐ GO:0005198	F	741	structural molecule activity					4.3	43	0.00095	-0.29	27	0.93	15	46	0	19	73	0
32	☐ GO:0016841	F	11	ammonia-lyase activity					3.9	1.6e+02	0.0059	-0.37	0.46	0.93	0.073	24	0.99	1.6	58	0.41
33	☐ GO:0003746	F	30	translation elongation factor activity					3.5	97	0.026	-0.3	15	0.93	13	1.2e+02	0	12	1.7e+02	0
34	☐ GO:0005737	C	1440	cytoplasm					4.1	37	0.0062	-1.8	17	0.59	17	41	0	20	60	0
35	☐ GO:0005576	C	230	extracellular region					3.6	52	0.028	13	2.5e+02	0	-0.7	21	0.6	-1.4	18	0.37
36	☐ GO:0019953	P	49	sexual reproduction					-0.22	21	1	16	6.3e+02	0	-1.5	14	0.3	-1.4	12	0.39
37	☐ GO:0071555	P	65	cell wall organization					-0.98	11	1	13	4.4e+02	0	0.18	24	0.94	-1.1	16	0.55
38	☐ GO:0000003	P	76	reproduction					-0.42	19	1	13	4e+02	0	-2.2	12	0.088	-1.9	10	0.18
39	☐ GO:0030036	P	42	actin cytoskeleton organization					-0.53	15	1	12	5.2e+02	0	-0.62	19	0.76	-1.1	14	0.59
40	☐ GO:0030029	P	42	actin filament-based process					-0.53	15	1	12	5.2e+02	0	-0.62	19	0.76	-1.1	14	0.59
41	☐ GO:0042545	P	30	cell wall modification					-1	2.6	1	11	5.5e+02	0	-1.2	14	0.46	-1.6	5.2	0.3
42	☐ GO:0007010	P	64	cytoskeleton organization					-0.64	15	1	10	3.6e+02	0	-0.56	20	0.79	-1.3	14	0.46
43	☐ GO:0071554	P	102	cell wall organization or biogenesis					2.2	50	0.3	9.9	2.8e+02	0	-0.85	19	0.63	-1.8	13	0.22
44	☐ GO:0071669	P	30	plant-type cell wall organization or biogenesis					-0.77	8.4	1	8	4.1e+02	9.1e-14	-0.47	19	0.81	-1.5	6.7	0.34
45	☐ GO:0009664	P	30	plant-type cell wall organization					-0.77	8.4	1	8	4.1e+02	9.1e-14	-0.47	19	0.81	-1.5	6.7	0.34
46	☐ GO:0009415	P	17	response to water					1.5	67	0.82	6	4.1e+02	1.1e-07	2.3	46	0.066	0.5	33	0.82
47	☐ GO:0006996	P	278	organelle organization					0.42	27	1	4.6	1e+02	0.00024	14	57	0	5.2	46	1.5e-06
48	☐ GO:0016043	P	488	cellular component organization					1.5	32	0.82	4.1	78	0.0019	15	52	0	6.3	44	2.7e-09
49	☐ GO:0009075	P	12	histidine family amino acid metabolic process					-0.32	14	1	4	3.3e+02	0.0034	-0.17	21	0.94	0.019	25	1

50	GO:0006547		P	12	histidine metabolic process	-0.32	14	1	4	3.3e+02	0.0034	-0.17	21	0.94	0.019	25	1
51	GO:0009628		P	756	response to abiotic stimulus	-0.56	22	1	3.8	66	0.0054	-3	18	0.011	-0.19	24	0.97
52	GO:0006950		P	1237	response to stress	2.1	31	0.36	3.7	57	0.0083	-3.3	19	0.0043	-0.37	24	0.89
53	GO:0050896		P	1404	response to stimulus	1.7	30	0.67	3.5	53	0.018	-3.6	19	0.0016	-1.1	23	0.55
54	GO:0042221		P	258	response to chemical stimulus	0.48	28	1	3.4	85	0.019	-1.2	20	0.46	-0.44	23	0.85
55	GO:0050826		P	705	response to freezing	-0.78	21	1	3.2	60	0.032	-3	18	0.0088	-0.038	25	0.99
56	GO:0048871		P	705	multicellular organismal homeostasis	-0.78	21	1	3.2	60	0.032	-3	18	0.0088	-0.038	25	0.99
57	GO:0042309		P	705	homoiothermy	-0.78	21	1	3.2	60	0.032	-3	18	0.0088	-0.038	25	0.99
58	GO:0009409		P	705	response to cold	-0.78	21	1	3.2	60	0.032	-3	18	0.0088	-0.038	25	0.99
59	GO:0001659		P	705	temperature homeostasis	-0.78	21	1	3.2	60	0.032	-3	18	0.0088	-0.038	25	0.99
60	GO:0009266		P	711	response to temperature stimulus	-0.8	21	1	3.1	60	0.036	-3.1	18	0.0083	-0.044	25	0.99
61	GO:0005975		P	775	carbohydrate metabolic process	2.3	34	0.26	3.1	58	0.041	4.5	29	3.9e-05	7.6	43	2.8e-13
62	GO:0043623	0043623	P	60	cellular protein complex assembly	2.6	63	0.15	3	1.3e+02	0.047	9.8	75	0	4.6	65	1.9e-05
63	GO:0030599		F	55	pectinesterase activity	-1.1	7.9	1	21	7.7e+02	0	-2.5	8.8	0.071	-2.3	3.7	0.1
64	GO:0004091		F	106	carboxylesterase activity	-1.2	11	1	15	4e+02	0	-2.7	12	0.045	-2.6	7.9	0.053
65	GO:0004857		F	87	enzyme inhibitor activity	0.81	35	1	14	4.2e+02	0	-2.7	11	0.044	-1	17	0.68
66	GO:0003779		F	79	actin binding	-0.41	19	1	12	3.9e+02	0	0.48	25	0.85	-0.81	19	0.75
67	GO:0008092		F	82	cytoskeletal protein binding	-0.46	19	1	12	3.7e+02	0	0.57	25	0.85	-0.84	19	0.75
68	GO:0004650		F	35	polygalacturonase activity	-1.1	3.5	1	12	5.5e+02	0	-1.9	9.3	0.23	-1.7	5.6	0.35
69	GO:0030234		F	229	enzyme regulator activity	0.42	28	1	9.4	1.9e+02	0	-0.53	21	0.85	-1.4	19	0.52
70	GO:0005507		F	73	copper ion binding	-1.2	8.6	1	6.2	2.2e+02	2.7e-08	-3	8.2	0.018	0.9	32	0.75
71	GO:0016788		F	597	hydrolase activity, acting on ester bonds	-0.5	22	1	5.8	91	4.4e-07	-3.1	18	0.016	-3.2	16	0.011
72	GO:0016835		F	72	carbon-oxygen lyase activity	0.37	30	1	4.9	1.8e+02	4.1e-05	1.1	28	0.6	4.5	61	9.4e-05
73	GO:0004553		F	312	hydrolase activity, hydrolyzing O-glycosyl compounds	2.4	40	0.33	4.6	97	0.00017	-0.12	22	0.99	0.28	26	0.89
74	GO:0016798		F	330	hydrolase activity, acting on glycosyl bonds	2.2	38	0.39	4.4	92	0.00048	-0.38	22	0.9	0.12	25	0.95
75	GO:0016161		F	12	beta-amylase activity	-0.46	9.1	1	4	3.3e+02	0.0025	-0.48	17	0.86	-0.34	18	0.88
76	GO:0016160		F	16	amylase activity	-0.42	13	1	3.4	2.5e+02	0.026	-0.61	16	0.83	-0.5	17	0.82
77	GO:0005337		F	10	nucleoside transmembrane transporter activity	-0.21	17	1	3.3	3e+02	0.035	-0.51	16	0.85	0.87	43	0.75
78	GO:0050825		F	705	ice binding	-0.78	21	1	3.2	60	0.043	-3	18	0.017	-0.038	25	0.98
79	GO:0050824		F	705	water binding	-0.78	21	1	3.2	60	0.043	-3	18	0.017	-0.038	25	0.98
80	GO:0044421		C	17	extracellular region part	-0.49	11	1	8.2	5.5e+02	2e-14	-1.5	7.7	0.23	-1.1	7	0.58
81	GO:0005618		C	66	cell wall	-1	9.8	1	7.1	2.6e+02	1e-10	1.3	29	0.3	-0.36	22	0.83
82	GO:0015629		C	116	actin cytoskeleton	-0.097	23	1	6.8	1.9e+02	6.3e-10	-0.023	23	0.98	-0.67	21	0.74
83	GO:0005856		C	208	cytoskeleton	1.1	34	1	6.7	1.5e+02	6.6e-10	5	37	3.8e-06	1.5	32	0.33
84	GO:0030312		C	84	external encapsulating structure	-1.2	9.8	1	6	2e+02	4.7e-08	0.73	26	0.59	-0.61	20	0.74
85	GO:0046821		C	12	extrachromosomal DNA	-0.51	7.6	1	4.3	3.5e+02	0.00042	-1.3	7.6	0.32	-0.64	12	0.74
86	GO:0005727		C	12	extrachromosomal circular DNA	-0.51	7.6	1	4.3	3.5e+02	0.00042	-1.3	7.6	0.32	-0.64	12	0.74
87	GO:0005874		C	50	microtubule	2.9	72	0.087	3.5	1.6e+02	0.0098	10	84	0	5.4	77	2.8e-07
88	GO:0034621		P	204	cellular macromolecular complex subunit organization	2.9	48	0.088	1.8	63	0.54	22	87	0	10	74	0
89	GO:0034622		P	188	cellular macromolecular complex assembly	2.7	47	0.13	2	68	0.36	22	89	0	10	74	0
90	GO:0065004		P	126	protein-DNA complex assembly	1.5	39	0.86	0.43	39	0.93	20	96	0	8.9	79	0
91	GO:0034728		P	126	nucleosome organization	1.5	39	0.86	0.43	39	0.93	20	96	0	8.9	79	0
92	GO:0031497		P	126	chromatin assembly	1.5	39	0.86	0.43	39	0.93	20	96	0	8.9	79	0
93	GO:0006334		P	126	nucleosome assembly	1.5	39	0.86	0.43	39	0.93	20	96	0	8.9	79	0
94	GO:0006323		P	127	DNA packaging	1.4	39	0.86	0.42	39	0.93	20	95	0	8.9	78	0
95	GO:0006333		P	140	chromatin assembly or disassembly	1.3	37	0.98	0.29	36	0.93	19	89	0	8.4	73	0
96	GO:0043933	0043933	P	319	macromolecular complex subunit organization	2.4	40	0.19	0.95	43	0.93	18	65	0	8.2	56	3.5e-15
97	GO:0065003		P	303	macromolecular complex assembly	2.2	39	0.3	1.1	45	0.93	18	66	0	7.8	55	8.9e-14
98	GO:0022607		P	315	cellular component assembly	2.1	38	0.36	0.97	44	0.93	18	64	0	7.5	54	6.6e-13
99	GO:0051276		P	179	chromosome organization	0.88	32	1	-0.053	28	0.98	17	77	0	6.9	60	4.2e-11
100	GO:0071103		P	174	DNA conformation change	1.1	34	1	0.02	30	0.99	17	77	0	8.7	70	0
101	GO:0006325		P	173	chromatin organization	0.94	33	1	-0.003	29	1	17	78	0	7.1	61	1.8e-11
102	GO:0044085		P	368	cellular component biogenesis	1.9	36	0.47	0.62	38	0.93	17	59	0	7.9	53	5.7e-14
103	GO:0006412		P	681	translation	2.5	36	0.17	-2.1	7.9	0.34	15	47	0	22	83	0
104	GO:0009987		P	7194	cellular process	1.1	26	1	-2.1	23	0.35	11	28	0	11	34	0
105	GO:0044237		P	5578	cellular metabolic process	1.2	26	1	-3.5	17	0.018	9.4	28	0	12	36	0
106	GO:0044267		P	2363	cellular protein metabolic process	0.67	26	1	-1.2	23	0.93	9.3	31	0	9.6	38	0
107	GO:0044249		P	2873	cellular biosynthetic process	1.1	27	1	-3.2	14	0.032	9	30	0	10	38	0
108	GO:0009058		P	3025	biosynthetic process	1.1	27	1	-3.2	14	0.032	8.8	29	0	11	38	0
109	GO:0034645		P	2180	cellular macromolecule biosynthetic process	0.023	25	1	-3.2	12	0.032	8.5	30	0	8.2	37	3.5e-15

110	GO:0007018	P	98	microtubule-based movement	1.2	39	1	2.1	84	0.36	8.5	58	0	3.1	46	0.0072
111	GO:0010467	P	2144	gene expression	0.22	25	1	-3.3	11	0.026	8.5	30	0	8.2	37	3.5e-15
112	GO:0009059	P	2183	macromolecule biosynthetic process	0.017	25	1	-3.2	12	0.032	8.5	30	0	8.2	37	6.9e-15
113	GO:0019538	P	2787	protein metabolic process	1	27	1	-1.6	21	0.79	8.4	29	0	8.8	36	0
114	GO:0044260	P	4281	cellular macromolecule metabolic process	-0.28	24	1	-3	17	0.052	8.3	28	0	5.7	31	1e-07
115	GO:0044238	P	6051	primary metabolic process	1.8	27	0.58	-2.1	22	0.35	8.2	27	4.6e-15	8.1	32	1e-14
116	GO:0006457	P	235	protein folding	2.4	43	0.19	-0.49	21	0.93	8.2	45	8.9e-15	6.5	54	6.3e-10
117	GO:0070271	P	189	protein complex biogenesis	1.5	37	0.84	0.91	47	0.93	8	47	1.7e-14	3.2	41	0.0053
118	GO:0006461	P	189	protein complex assembly	1.5	37	0.84	0.91	47	0.93	8	47	1.7e-14	3.2	41	0.0053
119	GO:0043170	P	4738	macromolecule metabolic process	0.92	26	1	-3	18	0.047	7.7	27	2e-13	5.2	30	9.3e-07
120	GO:0015986	P	64	ATP synthesis coupled proton transport	2.8	65	0.094	-0.47	14	0.93	7.5	61	1.4e-12	10	1.1e+02	0
121	GO:0015985	P	64	energy coupled proton transport, down electrochemical gradient	2.8	65	0.094	-0.47	14	0.93	7.5	61	1.4e-12	10	1.1e+02	0
122	GO:0006091	P	249	generation of precursor metabolites and energy	2.4	42	0.19	-0.46	22	0.93	7.4	42	1.6e-12	15	87	0
123	GO:0034220	P	66	ion transmembrane transport	2.9	65	0.094	-0.48	14	0.93	7.4	61	1.7e-12	10	1.1e+02	0
124	GO:0006596	P	12	polyamine biosynthetic process	1.6	78	0.72	1.7	1.6e+02	0.72	7.4	1.1e+02	1.8e-12	6.1	1.4e+02	7.2e-09
125	GO:0006595	P	12	polyamine metabolic process	1.6	78	0.72	1.7	1.6e+02	0.72	7.4	1.1e+02	1.8e-12	6.1	1.4e+02	7.2e-09
126	GO:0015992	P	76	proton transport	2.9	62	0.092	-0.34	19	0.93	7.4	58	2.2e-12	10	1e+02	0
127	GO:0006818	P	76	hydrogen transport	2.9	62	0.092	-0.34	19	0.93	7.4	58	2.2e-12	10	1e+02	0
128	GO:0007017	P	128	microtubule-based process	0.79	33	1	1.7	68	0.72	7	48	4.6e-11	2.4	39	0.062
129	GO:0044281	P	1146	small molecule metabolic process	2.9	34	0.09	-1.3	20	0.93	6.9	31	6.3e-11	11	48	0
130	GO:0042401	P	15	cellular biogenic amine biosynthetic process	1.4	67	0.86	1.4	1.2e+02	0.93	6.9	96	6.8e-11	5.7	1.2e+02	1e-07
131	GO:0006119	P	79	oxidative phosphorylation	2.6	59	0.15	-0.56	13	0.93	6.7	54	3e-10	9.5	97	0
132	GO:0051246	P	85	regulation of protein metabolic process	3	61	0.086	-0.47	16	0.93	6.7	53	3.1e-10	2.9	46	0.016
133	GO:0060348	P	15	bone development	0.6	42	1	-0.4	2.4	0.93	6.6	93	4.7e-10	3.6	88	0.0015
134	GO:0001503	P	15	ossification	0.6	42	1	-0.4	2.4	0.93	6.6	93	4.7e-10	3.6	88	0.0015
135	GO:0001501	P	15	skeletal system development	0.6	42	1	-0.4	2.4	0.93	6.6	93	4.7e-10	3.6	88	0.0015
136	GO:0044262	P	440	cellular carbohydrate metabolic process	0.62	28	1	-0.47	24	0.93	6.4	35	1.8e-09	8.7	53	0
137	GO:0048731	P	19	system development	0.5	38	1	-0.46	1.9	0.93	6.2	82	5.3e-09	3.1	73	0.0087
138	GO:0044265	P	254	cellular macromolecule catabolic process	2.4	42	0.19	-0.92	14	0.93	6	38	2.3e-08	6.4	52	1.6e-09
139	GO:0048856	P	20	anatomical structure development	0.45	36	1	-0.47	1.8	0.93	6	78	2.3e-08	3	70	0.012
140	GO:0006006	P	169	glucose metabolic process	1.4	37	0.91	-0.18	26	0.93	5.9	41	4.1e-08	11	83	0
141	GO:0030163	P	141	protein catabolic process	1.1	35	1	-1	7.5	0.93	5.9	43	4.3e-08	1.5	33	0.33
142	GO:0005996	P	213	monosaccharide metabolic process	1.2	34	1	-0.45	21	0.93	5.6	39	2.5e-07	10	71	0
143	GO:0031325	P	11	positive regulation of cellular metabolic process	1.6	81	0.71	-0.32	4.5	0.93	5.6	92	2.5e-07	2.9	84	0.014
144	GO:0010604	P	11	positive regulation of macromolecule metabolic process	1.6	81	0.71	-0.32	4.5	0.93	5.6	92	2.5e-07	2.9	84	0.014
145	GO:0009893	P	11	positive regulation of metabolic process	1.6	81	0.71	-0.32	4.5	0.93	5.6	92	2.5e-07	2.9	84	0.014
146	GO:0009141	P	141	nucleoside triphosphate metabolic process	1.9	43	0.47	-0.59	16	0.93	5.5	42	4.1e-07	7	65	3.4e-11
147	GO:0009206	P	140	purine ribonucleoside triphosphate biosynthetic process	1.9	43	0.47	-0.59	16	0.93	5.5	42	4.5e-07	7	65	2.8e-11
148	GO:0009205	P	140	purine ribonucleoside triphosphate metabolic process	1.9	43	0.47	-0.59	16	0.93	5.5	42	4.5e-07	7	65	2.8e-11
149	GO:0009201	P	140	ribonucleoside triphosphate biosynthetic process	1.9	43	0.47	-0.59	16	0.93	5.5	42	4.5e-07	7	65	2.8e-11
150	GO:0009199	P	140	ribonucleoside triphosphate metabolic process	1.9	43	0.47	-0.59	16	0.93	5.5	42	4.5e-07	7	65	2.8e-11
151	GO:0009145	P	140	purine nucleoside triphosphate biosynthetic process	1.9	43	0.47	-0.59	16	0.93	5.5	42	4.5e-07	7	65	2.8e-11
152	GO:0009144	P	140	purine nucleoside triphosphate metabolic process	1.9	43	0.47	-0.59	16	0.93	5.5	42	4.5e-07	7	65	2.8e-11
153	GO:0009142	P	140	nucleoside triphosphate biosynthetic process	1.9	43	0.47	-0.59	16	0.93	5.5	42	4.5e-07	7	65	2.8e-11
154	GO:0019318	P	190	hexose metabolic process	1.3	36	0.94	-0.3	24	0.93	5.4	39	5.3e-07	10	76	0
155	GO:0046034	P	133	ATP metabolic process	1.8	43	0.52	-0.58	16	0.93	5.4	42	7.9e-07	6.7	64	1.7e-10
156	GO:0006754	P	133	ATP biosynthetic process	1.8	43	0.52	-0.58	16	0.93	5.4	42	7.9e-07	6.7	64	1.7e-10
157	GO:0008152	P	7584	metabolic process	1.8	27	0.53	-1.9	24	0.48	5.3	25	9.1e-07	11	33	0
158	GO:0018193	P	12	peptidyl-amino acid modification	1.4	72	0.86	-0.35	3.4	0.93	5.3	85	1.3e-06	3.3	90	0.0037
159	GO:0009309	P	143	amine biosynthetic process	0.7	31	1	0.91	49	0.93	5.2	41	2.2e-06	4.8	52	1e-05
160	GO:0044248	P	329	cellular catabolic process	2.8	42	0.11	-1.2	13	0.93	5.1	34	2.5e-06	7.2	52	8.9e-12
161	GO:0042026	P	25	protein refolding	0.58	38	1	-0.55	0.96	0.93	5.1	65	3.2e-06	4.1	80	0.00021
162	GO:0009260	P	163	ribonucleotide biosynthetic process	1.7	40	0.64	-0.68	15	0.93	5	39	4.7e-06	6.5	59	7.9e-10
163	GO:0009259	P	163	ribonucleotide metabolic process	1.7	40	0.64	-0.68	15	0.93	5	39	4.7e-06	6.5	59	7.9e-10
164	GO:0009152	P	157	purine ribonucleotide biosynthetic process	1.7	40	0.63	-0.69	15	0.93	5	39	5e-06	6.5	60	6.3e-10
165	GO:0009150	P	157	purine ribonucleotide metabolic process	1.7	40	0.63	-0.69	15	0.93	5	39	5e-06	6.5	60	6.3e-10
166	GO:0048522	P	13	positive regulation of cellular process	1.4	70	0.86	-0.35	3.8	0.93	4.9	79	6.8e-06	2.5	71	0.045
167	GO:0048518	P	13	positive regulation of biological process	1.4	70	0.86	-0.35	3.8	0.93	4.9	79	6.8e-06	2.5	71	0.045
168	GO:0046365	P	125	monosaccharide catabolic process	1.3	38	0.98	-0.36	21	0.93	4.9	41	7.6e-06	8.4	76	0
169	GO:0019320	P	125	hexose catabolic process	1.3	38	0.98	-0.36	21	0.93	4.9	41	7.6e-06	8.4	76	0

170	GO:0006007		P	125	glucose catabolic process	1.3	38	0.98	-0.36	21	0.93	4.9	41	7.6e-06	8.4	76	0
171	GO:0006164		P	162	purine nucleotide biosynthetic process	1.7	40	0.67	-0.72	15	0.93	4.8	38	9.3e-06	6.4	59	1.5e-09
172	GO:0042398		P	28	cellular amino acid derivative biosynthetic process	0.99	46	1	1.4	97	0.93	4.8	60	1e-05	4.1	77	0.0002
173	GO:0006163		P	165	purine nucleotide metabolic process	1.6	39	0.71	-0.74	14	0.93	4.8	38	1.2e-05	6.3	58	2.7e-09
174	GO:0046164		P	130	alcohol catabolic process	1.3	38	0.94	-0.4	20	0.93	4.7	40	1.7e-05	8.3	74	0
175	GO:0044275		P	130	cellular carbohydrate catabolic process	1.3	38	0.94	-0.4	20	0.93	4.7	40	1.7e-05	8.3	74	0
176	GO:0046907		P	306	intracellular transport	1.2	32	1	-0.84	17	0.93	4.7	34	1.9e-05	1.5	31	0.35
177	GO:0045184		P	362	establishment of protein localization	1.3	32	0.98	-0.76	19	0.93	4.5	33	3.7e-05	1.3	30	0.44
178	GO:0015031		P	362	protein transport	1.3	32	0.98	-0.76	19	0.93	4.5	33	3.7e-05	1.3	30	0.44
179	GO:0006886		P	256	intracellular protein transport	1.3	34	0.98	-0.67	18	0.93	4.5	34	3.7e-05	1.4	31	0.36
180	GO:0015672		P	219	monovalent inorganic cation transport	1.7	38	0.64	-0.76	16	0.93	4.5	35	3.8e-05	7.7	60	1.3e-13
181	GO:0070727		P	261	cellular macromolecule localization	1.3	34	0.98	-0.7	18	0.93	4.5	34	4.5e-05	1.5	31	0.33
182	GO:0034613		P	261	cellular protein localization	1.3	34	0.98	-0.7	18	0.93	4.5	34	4.5e-05	1.5	31	0.33
183	GO:0006096		P	102	glycolysis	1.2	38	1	-0.2	24	0.93	4.5	41	5.5e-05	8.3	80	3.5e-15
184	GO:0009165		P	188	nucleotide biosynthetic process	1.4	36	0.87	-0.83	14	0.93	4.4	36	6.1e-05	5.8	54	4.3e-08
185	GO:0006519		P	386	cellular amino acid and derivative metabolic process	1.1	31	1	-0.32	25	0.93	4.4	32	6.1e-05	5.8	45	4.1e-08
186	GO:0008104		P	377	protein localization	1.2	32	1	-0.82	18	0.93	4.4	32	6.8e-05	1.3	29	0.46
187	GO:0055086		P	264	nucleobase, nucleoside and nucleotide metabolic process	1.2	33	1	-1.1	12	0.93	4.2	33	0.00014	5.3	47	6.3e-07
188	GO:0006415		P	13	translational termination	1.4	68	0.91	-0.36	3.3	0.93	4.2	71	0.00017	2.8	77	0.02
189	GO:0006575		P	65	cellular amino acid derivative metabolic process	1.1	40	1	0.51	46	0.93	4.2	44	0.00019	4.2	60	0.00011
190	GO:0044106		P	360	cellular amine metabolic process	0.91	30	1	-0.36	24	0.93	4.2	32	0.0002	5.5	44	2.6e-07
191	GO:0009117		P	236	nucleotide metabolic process	1.3	34	0.98	-1	12	0.93	4.1	34	0.00022	5.4	49	3.4e-07
192	GO:0006753		P	236	nucleoside phosphate metabolic process	1.3	34	0.98	-1	12	0.93	4.1	34	0.00022	5.4	49	3.4e-07
193	GO:0051649		P	360	establishment of localization in cell	0.98	30	1	-1	15	0.93	4.1	32	0.00022	1	29	0.59
194	GO:0006576		P	37	cellular biogenic amine metabolic process	0.85	40	1	0.63	56	0.93	4	50	0.00036	4.1	71	0.00018
195	GO:0051641		P	375	cellular localization	0.9	30	1	-1.1	14	0.93	4	31	0.00039	1	28	0.61
196	GO:0010608		P	11	posttranscriptional regulation of gene expression	1.3	68	0.99	-0.28	7.3	0.93	4	72	0.00039	2.8	82	0.02
197	GO:0006417		P	11	regulation of translation	1.3	68	0.99	-0.28	7.3	0.93	4	72	0.00039	2.8	82	0.02
198	GO:0044042		P	98	glucan metabolic process	-0.59	18	1	-0.29	22	0.93	3.9	39	0.00044	1.2	33	0.48
199	GO:0006073		P	98	cellular glucan metabolic process	-0.59	18	1	-0.29	22	0.93	3.9	39	0.00044	1.2	33	0.48
200	GO:0051128		P	12	regulation of cellular component organization	1.1	62	1	-0.35	3.1	0.93	3.9	69	0.00055	2.2	68	0.089
201	GO:0006066		P	316	alcohol metabolic process	0.86	30	1	-0.27	25	0.93	3.9	32	0.00055	8	55	2.3e-14
202	GO:0009250		P	74	glucan biosynthetic process	-0.27	21	1	-0.17	24	0.94	3.9	41	0.00058	1.5	37	0.34
203	GO:0051603		P	119	proteolysis involved in cellular protein catabolic process	0.59	31	1	-0.88	8.4	0.93	3.9	37	0.00058	0.52	28	0.82
204	GO:0044257		P	119	cellular protein catabolic process	0.59	31	1	-0.88	8.4	0.93	3.9	37	0.00058	0.52	28	0.82
205	GO:0032268		P	12	regulation of cellular protein metabolic process	1.2	64	1	-0.3	6.7	0.93	3.8	68	0.00072	2.6	76	0.03
206	GO:0009082		P	16	branched chain family amino acid biosynthetic process	0.4	36	1	-0.43	1.1	0.93	3.8	62	0.00084	1.9	56	0.19
207	GO:0043624	0043624	P	16	cellular protein complex disassembly	1.1	57	1	-0.4	3.1	0.93	3.7	61	0.001	2.6	69	0.033
208	GO:0043241	0043241	P	16	protein complex disassembly	1.1	57	1	-0.4	3.1	0.93	3.7	61	0.001	2.6	69	0.033
209	GO:0034623		P	16	cellular macromolecular complex disassembly	1.1	57	1	-0.4	3.1	0.93	3.7	61	0.001	2.6	69	0.033
210	GO:0032984		P	16	macromolecular complex disassembly	1.1	57	1	-0.4	3.1	0.93	3.7	61	0.001	2.6	69	0.033
211	GO:0022411		P	16	cellular component disassembly	1.1	57	1	-0.4	3.1	0.93	3.7	61	0.001	2.6	69	0.033
212	GO:0044282		P	167	small molecule catabolic process	2.1	43	0.36	-0.65	16	0.93	3.6	34	0.0014	7.9	66	3.9e-14
213	GO:0006397		P	27	mRNA processing	0.49	35	1	-0.45	6.9	0.93	3.6	51	0.0017	0.52	32	0.82
214	GO:0016071		P	29	mRNA metabolic process	0.46	34	1	-0.47	6.5	0.93	3.6	50	0.0017	0.48	31	0.83
215	GO:0044271		P	248	cellular nitrogen compound biosynthetic process	0.1	25	1	0.027	30	0.99	3.5	32	0.002	5.3	47	8.9e-07
216	GO:0044264		P	130	cellular polysaccharide metabolic process	-0.4	20	1	-0.37	21	0.93	3.5	35	0.0022	1.4	33	0.38
217	GO:0034641		P	486	cellular nitrogen compound metabolic process	0.57	27	1	-0.88	19	0.93	3.5	29	0.0022	6.2	44	3.9e-09
218	GO:0007264		P	173	small GTPase mediated signal transduction	1.2	35	1	0.74	44	0.93	3.5	34	0.0023	0.79	29	0.75
219	GO:0010033		P	34	response to organic substance	-0.22	20	1	-0.65	0.27	0.93	3.4	47	0.003	-0.57	18	0.81
220	GO:0009725		P	34	response to hormone stimulus	-0.22	20	1	-0.65	0.27	0.93	3.4	47	0.003	-0.57	18	0.81
221	GO:0009719		P	34	response to endogenous stimulus	-0.22	20	1	-0.65	0.27	0.93	3.4	47	0.003	-0.57	18	0.81
222	GO:0007275		P	49	multicellular organismal development	-0.02	24	1	-0.29	19	0.93	3.4	43	0.003	1	35	0.6
223	GO:0044283		P	322	small molecule biosynthetic process	2.2	38	0.33	0.032	30	0.98	3.4	31	0.0032	3.9	39	0.00055
224	GO:0006555		P	11	methionine metabolic process	0.82	53	1	-0.32	4.4	0.93	3.4	65	0.0035	3.4	95	0.0026
225	GO:0030244		P	32	cellulose biosynthetic process	0.21	29	1	-0.43	9.6	0.93	3.3	47	0.0038	2.4	53	0.059
226	GO:0030243		P	32	cellulose metabolic process	0.21	29	1	-0.43	9.6	0.93	3.3	47	0.0038	2.4	53	0.059
227	GO:0033692		P	106	cellular polysaccharide biosynthetic process	-0.099	23	1	-0.27	23	0.93	3.3	36	0.004	1.6	36	0.28
228	GO:0032502		P	50	developmental process	-0.045	24	1	-0.3	18	0.93	3.3	42	0.004	1	35	0.61
229	GO:0043436	0043436	P	495	oxoacid metabolic process	2.6	38	0.15	-1	17	0.93	3.3	29	0.0041	6.5	45	6.1e-10

230	GO:0019752		P	495	carboxylic acid metabolic process	2.6	38	0.15	-1	17	0.93	3.3	29	0.0041	6.5	45	6.1e-10
231	GO:0006082		P	496	organic acid metabolic process	2.6	38	0.15	-1.1	17	0.93	3.3	29	0.0042	6.5	45	6e-10
232	GO:0007242		P	185	intracellular signaling cascade	1.2	34	1	0.61	41	0.93	3.3	33	0.0043	0.58	28	0.81
233	GO:0009081		P	26	branched chain family amino acid metabolic process	0.32	32	1	-0.54	2	0.93	3.3	49	0.0047	1.6	47	0.28
234	GO:0042180		P	502	cellular ketone metabolic process	2.5	37	0.17	-1.1	17	0.93	3.2	29	0.006	6.4	44	1.1e-09
235	GO:0000271		P	109	polysaccharide biosynthetic process	-0.13	23	1	-0.3	22	0.93	3.2	35	0.006	1.5	35	0.32
236	GO:0022610		P	132	biological adhesion	0.4	28	1	0.22	34	0.93	3.1	34	0.0068	0.86	30	0.7
237	GO:0007155		P	132	cell adhesion	0.4	28	1	0.22	34	0.93	3.1	34	0.0068	0.86	30	0.7
238	GO:0008652		P	133	cellular amino acid biosynthetic process	0.23	27	1	0.42	39	0.93	3.1	34	0.0073	3.1	43	0.0079
239	GO:0051274		P	11	beta-glucan biosynthetic process	0.24	33	1	-0.27	8.3	0.93	3.1	61	0.008	0.087	27	0.99
240	GO:0051273		P	11	beta-glucan metabolic process	0.24	33	1	-0.27	8.3	0.93	3.1	61	0.008	0.087	27	0.99
241	GO:0006075		P	11	1,3-beta-glucan biosynthetic process	0.24	33	1	-0.27	8.3	0.93	3.1	61	0.008	0.087	27	0.99
242	GO:0006074		P	11	1,3-beta-glucan metabolic process	0.24	33	1	-0.27	8.3	0.93	3.1	61	0.008	0.087	27	0.99
243	GO:0006520		P	337	cellular amino acid metabolic process	0.61	28	1	-0.66	20	0.93	3	30	0.0092	4.8	43	7.4e-06
244	GO:0006413		P	45	translational initiation	0.97	41	1	-0.41	13	0.93	3	41	0.0097	1.9	44	0.16
245	GO:0006812		P	397	cation transport	1.2	32	1	-1.2	14	0.93	3	29	0.011	4.9	41	6.3e-06
246	GO:0006352		P	69	transcription initiation	-0.4	19	1	-0.79	4.5	0.93	3	37	0.011	0.56	29	0.81
247	GO:0042254		P	51	ribosome biogenesis	0.24	28	1	-0.75	2.2	0.93	2.9	39	0.015	3	53	0.01
248	GO:0022613		P	51	ribonucleoprotein complex biogenesis	0.24	28	1	-0.75	2.2	0.93	2.9	39	0.015	3	53	0.01
249	GO:0043632	0043632	P	102	modification-dependent macromolecule catabolic process	0.29	28	1	-0.79	8.9	0.93	2.9	34	0.015	-0.022	25	1
250	GO:0019941		P	102	modification-dependent protein catabolic process	0.29	28	1	-0.79	8.9	0.93	2.9	34	0.015	-0.022	25	1
251	GO:0006511		P	102	ubiquitin-dependent protein catabolic process	0.29	28	1	-0.79	8.9	0.93	2.9	34	0.015	-0.022	25	1
252	GO:0006108		P	20	malate metabolic process	0.88	47	1	-0.21	17	0.93	2.8	49	0.016	8.4	1.5e+02	0
253	GO:0051169		P	61	nuclear transport	0.81	36	1	0.073	32	0.98	2.8	38	0.016	0.97	33	0.62
254	GO:0006913		P	61	nucleocytoplasmic transport	0.81	36	1	0.073	32	0.98	2.8	38	0.016	0.97	33	0.62
255	GO:0034637		P	143	cellular carbohydrate biosynthetic process	-0.14	23	1	-0.4	21	0.93	2.8	32	0.017	1.6	34	0.28
256	GO:0016051		P	148	carbohydrate biosynthetic process	-0.17	23	1	-0.44	20	0.93	2.8	32	0.019	1.5	33	0.33
257	GO:0043648	0043648	P	56	dicarboxylic acid metabolic process	0.41	31	1	-0.5	12	0.93	2.8	38	0.019	5.3	73	7.3e-07
258	GO:0046483		P	347	heterocycle metabolic process	0.43	27	1	-0.69	20	0.93	2.7	29	0.022	6	47	1.3e-08
259	GO:0006811		P	483	ion transport	1.1	30	1	-1	17	0.93	2.6	28	0.028	4.3	38	8.9e-05
260	GO:0009086		P	10	methionine biosynthetic process	0.67	49	1	-0.31	3.7	0.93	2.5	55	0.04	2.5	79	0.041
261	GO:0006807		P	2524	nitrogen compound metabolic process	0.46	26	1	-3.3	12	0.026	2.5	25	0.044	-0.077	25	0.99
262	GO:0043094	0043094	P	17	cellular metabolic compound salvage	0.25	32	1	-0.34	7.9	0.93	2.4	47	0.047	1.4	48	0.37
263	GO:0006396		P	188	RNA processing	-0.23	23	1	-1.4	3.2	0.93	2.4	30	0.049	0.27	26	0.96
264	GO:0003735		F	484	structural constituent of ribosome	2	35	0.57	-1.8	8.5	0.86	14	48	0	23	94	0
265	GO:0003924		F	154	GTPase activity	3	52	0.14	1.8	67	0.85	11	60	0	7	63	4.7e-11
266	GO:0003676		F	2585	nucleic acid binding	0.13	25	1	-3.3	13	0.035	11	31	0	1.7	27	0.33
267	GO:0008135		F	87	translation factor activity, nucleic acid binding	2.8	59	0.19	-0.61	12	0.93	9.9	66	0	8.7	88	0
268	GO:0032561		F	289	guanyl ribonucleotide binding	2.4	41	0.33	1.5	52	0.93	9.1	45	0	5.6	47	4.4e-07
269	GO:0005525		F	289	GTP binding	2.4	41	0.33	1.5	52	0.93	9.1	45	0	5.6	47	4.4e-07
270	GO:0019001		F	299	guanyl nucleotide binding	2.3	40	0.36	1.4	51	0.93	9	44	0	5.4	46	1e-06
271	GO:0003723		F	404	RNA binding	1.2	31	1	-2	3.4	0.64	8	39	7.7e-14	7	49	4.8e-11
272	GO:0051082		F	150	unfolded protein binding	2.3	46	0.34	-1.1	5.9	0.93	7.2	47	2.4e-11	4.5	50	9.4e-05
273	GO:0016462		F	886	pyrophosphatase activity	1.1	29	1	-1	20	0.93	7.2	33	3e-11	3.5	33	0.0042
274	GO:0016817		F	908	hydrolase activity, acting on acid anhydrides	1	28	1	-1.1	20	0.93	7.1	32	5.8e-11	3.4	32	0.0066
275	GO:0005488		F	8563	binding	1.3	26	1	0.57	31	0.93	7	26	7.2e-11	2.2	27	0.12
276	GO:0016818		F	903	hydrolase activity, acting on acid anhydrides, in phosphorus-containing anhydrides	0.98	28	1	-1.1	20	0.93	7	32	8e-11	3.3	32	0.0076
277	GO:0019843		F	20	rRNA binding	1	51	1	-0.47	2.1	0.93	7	87	1.1e-10	9.2	1.6e+02	0
278	GO:0046961		F	29	proton-transporting ATPase activity, rotational mechanism	2.2	72	0.38	-0.18	21	0.93	6.6	74	9.6e-10	7.5	1.2e+02	2.2e-12
279	GO:0017111		F	856	nucleoside-triphosphatase activity	0.91	28	1	-1	20	0.93	6.6	32	9.6e-10	3	32	0.016
280	GO:0015078		F	105	hydrogen ion transmembrane transporter activity	2.6	53	0.25	-0.41	19	0.93	6.5	49	1.8e-09	10	93	0
281	GO:0046933		F	26	hydrogen ion transporting ATP synthase activity, rotational mechanism	1.9	66	0.76	-0.19	20	0.93	6.2	73	1.5e-08	7.5	1.2e+02	1.4e-12
282	GO:0019829		F	42	cation-transporting ATPase activity	1.7	54	0.97	-0.35	15	0.93	5.8	59	2.1e-07	5.9	87	6.2e-08
283	GO:0015077		F	128	monovalent inorganic cation transmembrane transporter activity	2.2	47	0.38	-0.55	17	0.93	5.6	43	4.1e-07	9.1	79	0
284	GO:0016620		F	23	oxidoreductase activity, acting on the aldehyde or oxo group of donors, NAD or NADP as acceptor	1.7	66	0.93	-0.22	17	0.93	5.2	68	3.9e-06	11	1.7e+02	0
285	GO:0051287		F	55	NAD or NADH binding	1.7	51	0.97	1.9	97	0.69	5	50	1.4e-05	11	1.2e+02	0
286	GO:0016903		F	32	oxidoreductase activity, acting on the aldehyde or oxo group of donors	1.6	57	1	-0.27	17	0.93	4.8	58	2.9e-05	9.5	1.4e+02	0
287	GO:0003677		F	1585	DNA binding	-1.5	20	1	-2.3	14	0.41	4.8	28	3.4e-05	-2.8	20	0.033
288	GO:0019904		F	12	protein domain specific binding	1.9	86	0.76	2.1	1.9e+02	0.48	4.8	79	3.4e-05	2.4	71	0.088
289	GO:0022890		F	174	inorganic cation transmembrane transporter activity	1.7	39	0.97	-0.82	13	0.93	4.7	37	4.8e-05	7.4	63	4.5e-12

290	GO:0035251		F	56	UDP-glucosyltransferase activity	0.31	29	1	-0.6	8.5	0.93	4.5	47	0.00011	5.1	71	4.4e-06
291	GO:0000166		F	2664	nucleotide binding	-0.099	24	1	0.52	32	0.93	4.4	26	0.00017	2.3	28	0.1
292	GO:0046527		F	68	glucosyltransferase activity	0.079	26	1	-0.7	7.3	0.93	4.4	45	0.00018	4.7	63	4e-05
293	GO:0032555		F	2410	purine ribonucleotide binding	-0.31	24	1	0.64	33	0.93	4.3	26	0.00021	0.73	26	0.77
294	GO:0032553		F	2410	ribonucleotide binding	-0.31	24	1	0.64	33	0.93	4.3	26	0.00021	0.73	26	0.77
295	GO:0004634		F	12	phosphopyruvate hydratase activity	0.92	55	1	0.22	46	0.93	4.2	73	0.00033	2.9	82	0.021
296	GO:0070003		F	29	threonine-type peptidase activity	1.3	52	1	-0.53	3.9	0.93	4.1	54	0.00043	1.9	49	0.24
297	GO:0004298		F	29	threonine-type endopeptidase activity	1.3	52	1	-0.53	3.9	0.93	4.1	54	0.00043	1.9	49	0.24
298	GO:0017076		F	2530	purine nucleotide binding	-0.35	24	1	0.4	31	0.93	3.9	26	0.0012	0.82	26	0.75
299	GO:0042625		F	98	ATPase activity, coupled to transmembrane movement of ions	1.1	37	1	-0.39	19	0.93	3.8	39	0.0016	3.5	49	0.0039
300	GO:0004427		F	28	inorganic diphosphatase activity	1	46	1	0.014	30	1	3.6	51	0.0037	2.8	60	0.033
301	GO:0016859		F	27	cis-trans isomerase activity	0.76	41	1	1.9	1.3e+02	0.68	3.5	50	0.0052	3.2	66	0.012
302	GO:0003755		F	27	peptidyl-prolyl cis-trans isomerase activity	0.76	41	1	1.9	1.3e+02	0.68	3.5	50	0.0052	3.2	66	0.012
303	GO:0016831		F	48	carboxy-lyase activity	0.79	38	1	0.48	47	0.93	3.4	43	0.0056	10	1.2e+02	0
304	GO:0015405		F	168	P-P-bond-hydrolysis-driven transmembrane transporter activity	0.84	32	1	-0.7	15	0.93	3.4	34	0.006	3.2	42	0.0099
305	GO:0015399		F	168	primary active transmembrane transporter activity	0.84	32	1	-0.7	15	0.93	3.4	34	0.006	3.2	42	0.0099
306	GO:0016986		F	29	transcription initiation factor activity	-0.44	15	1	-0.56	2.4	0.93	3.3	48	0.0071	1.6	45	0.41
307	GO:0016830		F	85	carbon-carbon lyase activity	0.61	32	1	0.32	38	0.93	3.3	38	0.0076	10	1e+02	0
308	GO:0004576		F	10	oligosaccharyl transferase activity	0.46	41	1	-0.34	1.3	0.93	3.2	65	0.011	0.8	42	0.75
309	GO:0003743		F	47	translation initiation factor activity	0.97	41	1	-0.44	13	0.93	3.2	42	0.011	2	45	0.2
310	GO:0003843		F	11	1,3-beta-glucan synthase activity	0.24	33	1	-0.27	8.3	0.93	3.1	61	0.016	0.087	27	0.96
311	GO:0000287		F	111	magnesium ion binding	1.2	37	1	-0.59	15	0.93	3.1	35	0.017	2.4	40	0.095
312	GO:0008194		F	86	UDP-glycosyltransferase activity	0.17	27	1	-0.52	15	0.93	3.1	36	0.017	3.6	51	0.0035
313	GO:0016820		F	138	hydrolase activity, acting on acid anhydrides, catalyzing transmembrane movement of substances	0.78	32	1	-0.7	14	0.93	3	33	0.018	2.8	41	0.027
314	GO:0008483		F	28	transaminase activity	0.62	38	1	-0.5	4.7	0.93	2.9	45	0.029	2.1	51	0.18
315	GO:0043492	0043492	F	133	ATPase activity, coupled to movement of substances	0.74	32	1	-0.67	14	0.93	2.9	33	0.029	2.7	41	0.035
316	GO:0042626		F	133	ATPase activity, coupled to transmembrane movement of substances	0.74	32	1	-0.67	14	0.93	2.9	33	0.029	2.7	41	0.035
317	GO:0016615		F	20	malate dehydrogenase activity	0.88	47	1	-0.21	17	0.93	2.8	49	0.03	8.4	1.5e+02	0
318	GO:0016832		F	10	aldehyde-lyase activity	0.96	59	1	0.79	94	0.93	2.8	60	0.03	8.8	2.1e+02	0
319	GO:0016853		F	234	isomerase activity	1.7	37	0.97	-0.22	26	0.93	2.8	30	0.033	3.8	42	0.0014
320	GO:0042623		F	270	ATPase activity, coupled	0.53	28	1	-1.3	8	0.93	2.8	30	0.035	1.6	31	0.41
321	GO:0032991		C	1357	macromolecular complex	2.8	33	0.12	-1.2	21	0.87	20	45	0	23	67	0
322	GO:0043232	0043232	C	936	intracellular non-membrane-bounded organelle	2.2	33	0.39	1.7	44	0.61	19	49	0	20	69	0
323	GO:0043228	0043228	C	936	non-membrane-bounded organelle	2.2	33	0.39	1.7	44	0.61	19	49	0	20	69	0
324	GO:0032993		C	123	protein-DNA complex	1.3	38	1	0.47	40	0.87	19	93	0	8.4	76	0
325	GO:0000786		C	123	nucleosome	1.3	38	1	0.47	40	0.87	19	93	0	8.4	76	0
326	GO:0044424		C	3090	intracellular part	2.4	30	0.29	-1	24	0.87	17	35	0	16	44	0
327	GO:0000785		C	169	chromatin	0.85	32	1	0.096	31	0.94	16	75	0	7.3	63	1.6e-12
328	GO:0044427		C	181	chromosomal part	0.72	31	1	-0.002	29	1	16	72	0	6.9	60	2.3e-11
329	GO:0005622		C	3688	intracellular	2	28	0.46	-0.85	26	0.87	16	33	0	14	40	0
330	GO:0005694		C	233	chromosome	0.5	28	1	-0.33	24	0.87	15	64	0	7.2	57	4.3e-12
331	GO:0043229	0043229	C	2373	intracellular organelle	1.9	29	0.46	-0.67	26	0.87	15	36	0	13	43	0
332	GO:0043226	0043226	C	2373	organelle	1.9	29	0.46	-0.67	26	0.87	15	36	0	13	43	0
333	GO:0044446		C	682	intracellular organelle part	1.4	31	1	-0.46	25	0.87	15	46	0	12	56	0
334	GO:0044422		C	682	organelle part	1.4	31	1	-0.46	25	0.87	15	46	0	12	56	0
335	GO:0005840		C	489	ribosome	2.1	35	0.39	-1.8	8.5	0.59	14	48	0	23	94	0
336	GO:0030529		C	534	ribonucleoprotein complex	2.1	35	0.39	-1.9	8.5	0.57	14	47	0	22	88	0
337	GO:0044444		C	1059	cytoplasmic part	3	35	0.087	-2.2	11	0.26	13	40	0	18	63	0
338	GO:0044464		C	5557	cell part	1.7	27	0.67	-0.092	29	0.94	11	29	0	9.6	34	0
339	GO:0005623		C	5557	cell	1.7	27	0.67	-0.092	29	0.94	11	29	0	9.6	34	0
340	GO:0015630		C	65	microtubule cytoskeleton	2.4	59	0.29	2.9	1.2e+02	0.076	9	69	0	4.3	61	5.9e-05
341	GO:0043234	0043234	C	718	protein complex	1.6	31	0.74	-0.34	26	0.87	8.6	36	0	10	50	0
342	GO:0043231	0043231	C	1596	intracellular membrane-bounded organelle	0.82	27	1	-2.1	16	0.35	8.1	31	4.9e-15	0.066	25	0.97
343	GO:0043227	0043227	C	1610	membrane-bounded organelle	0.79	27	1	-2.1	16	0.34	8	31	1.3e-14	1.1	27	0.57
344	GO:0044430		C	83	cytoskeletal part	2	49	0.46	2.3	96	0.26	7.8	58	3.5e-14	3.4	50	0.0022
345	GO:0016469		C	67	proton-transporting two-sector ATPase complex	2.9	66	0.087	-0.49	14	0.87	7.5	60	6.1e-13	10	1.1e+02	0
346	GO:0033178		C	36	proton-transporting two-sector ATPase complex, catalytic domain	2.1	65	0.39	-0.24	19	0.89	6.7	69	9.9e-11	7.9	1.1e+02	2.2e-14
347	GO:0005634		C	1190	nucleus	-0.49	23	1	-1.6	17	0.79	6.6	31	2.2e-10	-2.2	21	0.078
348	GO:0033279		C	67	ribosomal subunit	1	39	1	-0.75	5.5	0.87	5.9	52	2.3e-08	9.4	1e+02	0
349	GO:0033180		C	11	proton-transporting V-type ATPase, V1 domain	1.8	88	0.54	-0.26	9.2	0.89	5.5	91	2.4e-07	4.1	1.1e+02	0.00019

350	GO:0033176	C	29	proton-transporting V-type ATPase complex	2.1	70	0.39	-0.41	9.3	0.87	5.4	64	3.2e-07	4.1	77	0.00014
351	GO:0015935	C	39	small ribosomal subunit	1.2	46	1	-0.54	6.9	0.87	5	56	3.1e-06	7.7	1.1e+02	1.1e-13
352	GO:0000502	C	35	proteasome complex	1.4	52	1	-0.59	3.4	0.87	4.8	56	8.7e-06	2.1	49	0.11
353	GO:0045259	C	29	proton-transporting ATP synthase complex	1.5	57	0.88	-0.26	17	0.89	4.3	56	9.2e-05	10	1.5e+02	0
354	GO:0000015	C	12	phosphopyruvate hydratase complex	0.92	55	1	0.22	46	0.89	4.2	73	0.00013	2.9	82	0.012
355	GO:0005839	C	29	proteasome core complex	1.3	52	1	-0.53	3.9	0.87	4.1	54	0.00017	1.9	49	0.16
356	GO:0045261	C	19	proton-transporting ATP synthase complex, catalytic core F(1)	0.94	49	1	-0.1	23	0.94	3.8	59	0.00064	6.3	1.2e+02	1.5e-09
357	GO:0005783	C	112	endoplasmic reticulum	3.2	59	0.058	-0.83	9	0.87	3.8	37	0.00071	1.4	34	0.39
358	GO:0033177	C	29	proton-transporting two-sector ATPase complex, proton-transporting domain	2	67	0.46	-0.44	8.3	0.87	3.7	51	0.00092	6.9	1.1e+02	2.1e-11
359	GO:0044455	C	17	mitochondrial membrane part	1.2	57	1	-0.025	28	0.99	3.4	57	0.003	3.3	80	0.0031
360	GO:0005794	C	55	Golgi apparatus	0.63	34	1	-0.63	7.1	0.87	3.4	41	0.0031	1.2	36	0.48
361	GO:0046930	C	11	pore complex	0.56	44	1	-0.34	2.6	0.87	3.4	65	0.0031	1	45	0.64
362	GO:0005643	C	11	nuclear pore	0.56	44	1	-0.34	2.6	0.87	3.4	65	0.0031	1	45	0.64
363	GO:0015934	C	28	large ribosomal subunit	0.19	29	1	-0.53	3.4	0.87	3.2	48	0.0052	5.5	95	2.1e-07
364	GO:0005635	C	12	nuclear envelope	0.53	42	1	-0.35	2.9	0.87	3.2	60	0.0061	0.91	43	0.7
365	GO:0048475	C	56	coated membrane	0.51	32	1	-0.69	5.3	0.87	3.1	40	0.0063	0.93	33	0.69
366	GO:0030117	C	56	membrane coat	0.51	32	1	-0.69	5.3	0.87	3.1	40	0.0063	0.93	33	0.69
367	GO:0000148	C	11	1,3-beta-glucan synthase complex	0.24	33	1	-0.27	8.3	0.89	3.1	61	0.0072	0.087	27	0.97
368	GO:0044459	C	58	plasma membrane part	1.1	41	1	0.9	60	0.87	3	39	0.0085	0.87	33	0.73
369	GO:0044445	C	59	cytosolic part	0.36	30	1	-0.49	13	0.87	3	39	0.0092	2.3	45	0.076
370	GO:0005829	C	59	cytosol	0.36	30	1	-0.49	13	0.87	3	39	0.0092	2.3	45	0.076
371	GO:0031967	C	148	organelle envelope	0.27	27	1	-0.54	18	0.87	3	33	0.0099	2.5	39	0.038
372	GO:0005886	C	68	plasma membrane	0.95	38	1	0.73	52	0.87	3	38	0.01	1.5	37	0.37
373	GO:0031988	C	37	membrane-bounded vesicle	0.33	31	1	-0.57	4.8	0.87	2.8	42	0.016	0.62	32	0.74
374	GO:0016023	C	37	cytoplasmic membrane-bounded vesicle	0.33	31	1	-0.57	4.8	0.87	2.8	42	0.016	0.62	32	0.74
375	GO:0033179	C	16	proton-transporting V-type ATPase, V0 domain	1.2	60	1	-0.3	10	0.88	2.6	50	0.026	2	59	0.13
376	GO:0019773	C	12	proteasome core complex, alpha-subunit complex	1	58	1	-0.37	1.7	0.87	2.6	54	0.028	1.3	49	0.47
377	GO:0044429	C	146	mitochondrial part	0.2	26	1	-0.53	18	0.87	2.5	31	0.034	2.5	39	0.042
378	GO:0030118	C	21	clathrin coat	0.44	36	1	-0.45	4	0.87	2.5	45	0.037	0.84	37	0.74
379	GO:0012505	C	84	endomembrane system	0.19	27	1	-0.86	5	0.87	2.5	34	0.037	0.072	25	0.97
380	GO:0005798	C	14	Golgi-associated vesicle	0.35	35	1	-0.29	9.3	0.88	2.5	50	0.037	1	43	0.64
381	GO:0031975	C	167	envelope	0.06	25	1	-0.67	16	0.87	2.5	31	0.037	2.2	36	0.085
382	GO:0031982	C	41	vesicle	0.28	30	1	-0.61	4.4	0.87	2.4	38	0.039	0.5	30	0.75
383	GO:0031410	C	41	cytoplasmic vesicle	0.28	30	1	-0.61	4.4	0.87	2.4	38	0.039	0.5	30	0.75
384	GO:0005739	C	167	mitochondrion	0.088	25	1	-0.63	17	0.87	2.4	30	0.041	2.8	39	0.019
385	GO:0031966	C	120	mitochondrial membrane	0.25	27	1	-0.34	21	0.87	2.4	32	0.041	2.5	40	0.046
386	GO:0030135	C	33	coated vesicle	0.27	30	1	-0.54	4.8	0.87	2.3	40	0.047	0.47	30	0.76
387	GO:0015979	P	54	photosynthesis	-0.58	15	1	-0.81	0.63	0.93	-3.1	5.3	0.008	21	2.2e+02	0
388	GO:0071704	P	11	organic substance metabolic process	0.025	25	1	-0.34	2.8	0.93	0.038	23	0.99	13	3e+02	0
389	GO:0015977	P	11	carbon fixation	0.025	25	1	-0.34	2.8	0.93	0.038	23	0.99	13	3e+02	0
390	GO:0005985	P	14	sucrose metabolic process	0.15	29	1	-0.29	9.5	0.93	1.3	37	0.4	6.6	1.4e+02	3.6e-10
391	GO:0055114	P	994	oxidation reduction	2.7	34	0.13	0.47	33	0.93	-1.4	21	0.33	6.3	38	2.2e-09
392	GO:0046356	P	21	acetyl-CoA catabolic process	0.39	34	1	-0.18	19	0.93	1.6	37	0.25	5.4	1e+02	3.4e-07
393	GO:0009109	P	21	coenzyme catabolic process	0.39	34	1	-0.18	19	0.93	1.6	37	0.25	5.4	1e+02	3.4e-07
394	GO:0006099	P	21	tricarboxylic acid cycle	0.39	34	1	-0.18	19	0.93	1.6	37	0.25	5.4	1e+02	3.4e-07
395	GO:0006084	P	21	acetyl-CoA metabolic process	0.39	34	1	-0.18	19	0.93	1.6	37	0.25	5.4	1e+02	3.4e-07
396	GO:0015995	P	11	chlorophyll biosynthetic process	-0.36	12	1	-0.37	0.44	0.93	-0.86	12	0.63	5.3	1.3e+02	8.5e-07
397	GO:0015994	P	11	chlorophyll metabolic process	-0.36	12	1	-0.37	0.44	0.93	-0.86	12	0.63	5.3	1.3e+02	8.5e-07
398	GO:0051187	P	23	cofactor catabolic process	0.35	33	1	-0.22	17	0.93	1.4	35	0.34	5.2	98	1.1e-06
399	GO:0009060	P	25	aerobic respiration	0.57	38	1	-0.25	17	0.93	2.3	42	0.06	4.9	91	6.2e-06
400	GO:0045333	P	43	cellular respiration	0.68	36	1	-0.38	14	0.93	2.2	36	0.089	4.1	67	0.00018
401	GO:0033014	P	40	tetrapyrrole biosynthetic process	-0.68	12	1	-0.69	1.1	0.93	-1.4	13	0.33	3.9	67	0.00038
402	GO:0033013	P	42	tetrapyrrole metabolic process	-0.68	12	1	-0.7	1	0.93	-1.5	13	0.3	3.9	65	0.00054
403	GO:0016137	P	37	glycoside metabolic process	-0.15	22	1	-0.51	7.5	0.93	0.17	24	0.94	3.4	63	0.0029
404	GO:0005984	P	37	disaccharide metabolic process	-0.15	22	1	-0.51	7.5	0.93	0.17	24	0.94	3.4	63	0.0029
405	GO:0051186	P	211	cofactor metabolic process	-0.17	23	1	-1.3	5.5	0.93	-1.4	19	0.33	3.3	40	0.0046
406	GO:0009311	P	39	oligosaccharide metabolic process	-0.17	21	1	-0.53	7.2	0.93	0.14	24	0.95	3.3	60	0.0049
407	GO:0015980	P	65	energy derivation by oxidation of organic compounds	0.21	27	1	0.04	31	0.98	2	33	0.13	3.2	52	0.0053
408	GO:0006779	P	32	porphyrin biosynthetic process	-0.6	12	1	-0.62	0.96	0.93	-1.3	13	0.4	3.2	63	0.0063
409	GO:0000096	P	23	sulfur amino acid metabolic process	1.1	52	1	-0.48	3.2	0.93	2.1	41	0.096	3.2	70	0.0064

410	GO:0006778	P	34	porphyrin metabolic process	-0.6	13	1	-0.64	0.95	0.93	-1.4	13	0.36	3.1	61	0.0082
411	GO:0022900	P	35	electron transport chain	0.54	35	1	-0.48	8.1	0.93	0.35	25	0.87	3.1	60	0.0087
412	GO:0046148	P	32	pigment biosynthetic process	-0.36	17	1	0.037	31	0.98	-0.51	19	0.8	2.9	60	0.014
413	GO:0042440	P	34	pigment metabolic process	-0.36	17	1	-0.0018	29	1	-0.6	18	0.77	2.8	58	0.018
414	GO:0046394	P	219	carboxylic acid biosynthetic process	2.4	43	0.19	-0.059	28	0.98	2.1	29	0.096	2.8	38	0.019
415	GO:0016053	P	219	organic acid biosynthetic process	2.4	43	0.19	-0.059	28	0.98	2.1	29	0.096	2.8	38	0.019
416	GO:0018130	P	100	heterocycle biosynthetic process	-0.56	18	1	0.48	42	0.93	-0.16	22	0.94	2.8	44	0.02
417	GO:0006732	P	108	coenzyme metabolic process	0.17	26	1	-0.87	7.4	0.93	0.49	25	0.8	2.8	43	0.022
418	GO:0009084	P	29	glutamine family amino acid biosynthetic process	-0.033	24	1	-0.46	6.9	0.93	1.2	32	0.45	2.7	59	0.026
419	GO:0055085	P	595	transmembrane transport	-0.3	23	1	-1.2	16	0.93	-0.66	22	0.73	2.6	32	0.031
420	GO:0006730	P	47	one-carbon metabolic process	0.74	37	1	-0.49	11	0.93	2.2	36	0.086	2.6	50	0.033
421	GO:0009069	P	32	serine family amino acid metabolic process	0.34	31	1	-0.16	22	0.94	0.92	29	0.59	2.6	56	0.033
422	GO:0000097	P	20	sulfur amino acid biosynthetic process	1.1	52	1	-0.45	2.9	0.93	1.6	38	0.25	2.6	63	0.037
423	GO:0006541	P	21	glutamine metabolic process	0.14	28	1	-0.31	12	0.93	0.79	30	0.66	2.6	62	0.037
424	GO:0006544	P	14	glycine metabolic process	-0.063	23	1	-0.35	5.2	0.93	1.6	40	0.26	2.5	69	0.048
425	GO:0016829	F	229	lyase activity	1.6	37	1	3.1	82	0.052	2.3	29	0.12	9.1	66	0
426	GO:0004618	F	10	phosphoglycerate kinase activity	0.25	33	1	-0.32	2.6	0.93	1	36	0.65	6.5	1.6e+02	1.8e-09
427	GO:0048037	F	434	cofactor binding	1.3	32	1	-0.24	26	0.93	1.9	27	0.23	6.5	46	1.8e-09
428	GO:0004470	F	11	malic enzyme activity	0.52	42	1	-0.067	24	0.98	1.9	46	0.26	6.2	1.5e+02	1.2e-08
429	GO:0051537	F	23	2 iron, 2 sulfur cluster binding	0.18	29	1	-0.38	8.8	0.93	-1	14	0.65	5.9	1.1e+02	8e-08
430	GO:0016491	F	1202	oxidoreductase activity	2.2	32	0.39	0.47	33	0.93	-1.8	21	0.27	5.8	36	9.9e-08
431	GO:0050662	F	320	coenzyme binding	1.1	32	1	-0.49	22	0.93	0.94	25	0.69	5.5	46	4.4e-07
432	GO:0016616	F	184	oxidoreductase activity, acting on the CH-OH group of donors, NAD or NADP as acceptor	0.75	31	1	0.17	33	0.94	1.7	28	0.32	4.7	48	4e-05
433	GO:0016836	F	54	hydro-lyase activity	0.14	27	1	-0.43	14	0.93	1.4	30	0.49	4.6	67	6.1e-05
434	GO:0008324	F	447	cation transmembrane transporter activity	0.64	28	1	-1.3	13	0.93	1.8	26	0.27	4.4	39	0.00011
435	GO:0016614	F	202	oxidoreductase activity, acting on CH-OH group of donors	0.59	29	1	0.28	34	0.93	1.2	26	0.56	4.3	45	0.00023
436	GO:0016165	F	11	lipoxygenase activity	2	94	0.59	-0.37	0.35	0.93	1.7	43	0.36	4.3	1.1e+02	0.00024
437	GO:0005215	F	1049	transporter activity	0.41	26	1	-0.88	22	0.93	0.79	24	0.73	4	33	0.00072
438	GO:0051540	F	95	metal cluster binding	1	36	1	-0.82	7.4	0.93	0.5	25	0.85	3.9	52	0.00094
439	GO:0051536	F	95	iron-sulfur cluster binding	1	36	1	-0.82	7.4	0.93	0.5	25	0.85	3.9	52	0.00094
440	GO:0015075	F	575	ion transmembrane transporter activity	0.56	27	1	-1.2	17	0.93	1.6	26	0.36	3.7	35	0.0017
441	GO:0016774	F	28	phosphotransferase activity, carboxyl group as acceptor	-0.27	19	1	-0.54	2.9	0.93	0.87	30	0.71	3.5	70	0.0042
442	GO:0022891	F	659	substrate-specific transmembrane transporter activity	0.51	27	1	-0.73	22	0.93	1.2	25	0.56	3.3	34	0.0073
443	GO:0022892	F	737	substrate-specific transporter activity	0.48	27	1	-0.91	21	0.93	1.5	25	0.45	3.3	33	0.0073
444	GO:0016681	F	15	oxidoreductase activity, acting on diphenols and related substances as donors, cytochrome as acceptor	0.15	29	1	-0.16	18	0.94	0.057	23	0.99	3.2	81	0.0099
445	GO:0008121	F	15	ubiquinol-cytochrome-c reductase activity	0.15	29	1	-0.16	18	0.94	0.057	23	0.99	3.2	81	0.0099
446	GO:0022857	F	731	transmembrane transporter activity	0.47	26	1	-0.78	22	0.93	0.78	24	0.73	3.1	33	0.016
447	GO:0003918	F	34	DNA topoisomerase (ATP-hydrolyzing) activity	-0.034	24	1	-0.53	5.8	0.93	1.1	30	0.6	3	60	0.018
448	GO:0016776	F	25	phosphotransferase activity, phosphate group as acceptor	0.095	27	1	-0.27	15	0.93	0.73	29	0.76	3	65	0.018
449	GO:0016676	F	11	oxidoreductase activity, acting on heme group of donors, oxygen as acceptor	0.95	57	1	0.24	48	0.93	2.5	54	0.073	3	86	0.018
450	GO:0016675	F	11	oxidoreductase activity, acting on heme group of donors	0.95	57	1	0.24	48	0.93	2.5	54	0.073	3	86	0.018
451	GO:0015002	F	11	heme-copper terminal oxidase activity	0.95	57	1	0.24	48	0.93	2.5	54	0.073	3	86	0.018
452	GO:0004129	F	11	cytochrome-c oxidase activity	0.95	57	1	0.24	48	0.93	2.5	54	0.073	3	86	0.018
453	GO:0051213	F	22	dioxygenase activity	1.5	60	1	-0.47	3.2	0.93	1.2	34	0.55	3	67	0.02
454	GO:0003824	F	6876	catalytic activity	0.85	26	1	1.2	33	0.93	-0.68	22	0.78	2.9	27	0.02
455	GO:0016702	F	20	oxidoreductase activity, acting on single donors with incorporation of molecular oxygen, incorporation of two atoms of oxygen	1.4	61	1	-0.45	3.4	0.93	1	32	0.64	2.9	69	0.023
456	GO:0016701	F	27	oxidoreductase activity, acting on single donors with incorporation of molecular oxygen	1.2	51	1	-0.45	7	0.93	0.35	26	0.92	2.8	61	0.033
457	GO:0009521	C	33	photosystem	-0.32	18	1	-0.63	0.62	0.87	-2.8	2.3	0.014	25	3.2e+02	0
458	GO:0009579	C	45	thylakoid	-0.43	17	1	-0.74	0.53	0.87	-2.8	5.4	0.015	24	2.7e+02	0
459	GO:0034357	C	44	photosynthetic membrane	-0.4	18	1	-0.73	0.54	0.87	-2.8	5.5	0.017	24	2.7e+02	0
460	GO:0009522	C	14	photosystem I	-0.058	23	1	-0.42	0.36	0.87	-2	1.1	0.1	21	4.1e+02	0
461	GO:0044436	C	28	thylakoid part	-0.46	15	1	-0.59	0.37	0.87	-1.9	8	0.12	17	2.4e+02	0
462	GO:0009523	C	18	photosystem II	-0.34	15	1	-0.46	0.85	0.87	-2	3.3	0.099	15	2.6e+02	0
463	GO:0009654	C	14	oxygen evolving complex	-0.35	14	1	-0.42	0.47	0.87	-1.7	4	0.17	14	2.8e+02	0
464	GO:0019898	C	12	extrinsic to membrane	-0.29	15	1	-0.39	0.13	0.87	-1.4	6.1	0.28	13	2.7e+02	0
465	GO:0042651	C	14	thylakoid membrane	-0.26	16	1	-0.42	0.28	0.87	-0.97	12	0.46	11	2.3e+02	0
466	GO:0045263	C	10	proton-transporting ATP synthase complex, coupling factor F(o)	1.3	72	1	-0.3	4.7	0.88	2	49	0.093	8.4	2e+02	0
467	GO:0044425	C	1181	membrane part	0.94	28	1	-0.63	25	0.87	1.8	25	0.15	7.3	39	1.8e-12
468	GO:0031090	C	213	organelle membrane	0.14	26	1	-0.87	14	0.87	2.3	29	0.051	4.6	46	1.5e-05

469	GO:0016020	C	2254	membrane	0.6	26	1	-1.2	23	0.87	-0.53	22	0.66	3.6	30	0.0014
470	GO:0009536	C	33	plastid	-0.63	12	1	-0.61	1.5	0.87	-2	8.2	0.097	2.5	55	0.04
