# Supplementary material for: Identification of genes specifically or preferentially expressed in maize silk reveals similarity and diversity in transcript abundance of different dry stigmas
Source: BMC Genomics. 2012 Jul 2;13:294. doi: 10.1186/1471-2164-13-294 (PMC3416702; doi:10.1186/1471-2164-13-294)
Supplement: Additional file 7 — A list of real-time PCR primers used in this study. [file 1471-2164-13-294-S7.doc]

**Additional file 7. A list of the qRT-PCR primers used in this study.**

| Description | Name | Primer sequence |
| --- | --- | --- |
| Hypothetical protein | GRMZM2G137435-RL | AATCCTTCCAACCTCCTC |
|  | GRMZM2G137435-RR | GATGAAACGAATCCTTGTG |
| Expressed protein | GRMZM2G060940-RL | TCAGCACCAGGAAGAACAG |
|  | GRMZM2G060940-RR | AGCAGTAGCAGTAGTTAGGC |
| Esterase PIR7B | GRMZM2G428987-RL | CAGCAGAAGGTGACGATGTG |
|  | GRMZM2G428987-RR | CGTTGGCGAGGACTACCG |
| EF hand family protein | GRMZM2G436947-RL | TCACTACTTGCCTGCCACTC |
|  | GRMZM2G436947-RR | GCTGCTCTGCTTTGCTTCC |
| Expressed protein | GRMZM2G306028-RL | GCCAGTACCAGATCCAAGAAG |
|  | GRMZM2G306028-RR | GTGACGAAGCAACCGAAGG |
| Expressed protein | GRMZM2G439422-RL | ACCCGCACACCCACGCTTC |
|  | GRMZM2G439422-RR | GCCACGACGACGAGGAGGAG |
| No information | GRMZM2G093313-RL | TGCTCAACAATTCCATATCAAG |
|  | GRMZM2G093313-RR | CTTATCCAGTAGTAGTCCAACC |
| Hypothetical protein | GRMZM2G148800-RL | GTCGGGAAGGCGTTGCTG |
|  | GRMZM2G148800-RR | ATGTCTGGGAGAAGATGATCG |
| Hypothetical protein | GRMZM2G128549-RL | CGATGCCGAGGAGGTGAC |
|  | GRMZM2G128549-RR | TCCAGCGTATCCGACAGG |
| Expressed protein | GRMZM2G439422-RL | ACCCGCACACCCACGCTTC |
|  | GRMZM2G439422-RR | GCCACGACGACGAGGAGGAG |
| Rab GDP dissociation inhibitor alpha | GRMZM2G084440-RL | AGCGTATGGAGTCACTTCTG |
|  | GRMZM2G084440-RR | ACCTTCTTCACCTTGTCTGG |
| No information | GRMZM2G343437-RL | GCCCTACAAGTCCAGCAG |
|  | GRMZM2G343437-RR | TCGTTCTCCTCCTTGATGG |
| Hypothetical protein | GRMZM2G108624-RL | CCATCTCATCGTCTCCTG |
|  | GRMZM2G108624-RR | TCCACCTAAAATAAAATCCATACA |
| No information | GRMZM2G375975-RL | TGCGACAACATACTTCCTTACC |
|  | GRMZM2G375975-RR | TGGGCTCCTCATCTTTCTCTC |
| Copper transporter 1 | GRMZM2G042412-RL | ATTCGGTGGGTTTGTCTATGTC |
|  | GRMZM2G042412-RR | ATAGGATACAGCCAAGAGATTCAC |
| Expressed protein | GRMZM2G154936-RL | CCAGCCGTCAGCGTAGTG |
|  | GRMZM2G154936-RR | ATATAAGCAAGGAAGCGACAGAAG |
| Hypothetical protein | GRMZM2G312661-RL | CTCAACGAACTCCAGAAATCATC |
|  | GRMZM2G312661-RR | GCGACGGCTTAGCAATGG |
| Hypothetical protein | GRMZM2G010855-RL | AAATCCCACTAGCGAAAGG |
|  | GRMZM2G010855-RR | ACAGAGATGCCACCAAGG |
| No information | GRMZM2G889467-RL | TGAGGAGAAGAAGAGGGAGAAGAG |
|  | GRMZM2G889467-RR | ACGACGAGGAGGTTGGAAGG |
| Hypothetical protein | GRMZM2G137211-RL | GTGATGTGGAAAGAGATGGTAAAC |
|  | GRMZM2G137211-RR | TTTGGAAATGCTTCTTGCTGAG |
| Carbonic anhydrase | GRMZM2G121878-RL | AAGAGGAGGAGAAGAAGAAGAAG |
|  | GRMZM2G121878-RR | GGAGGAGGTGGAGGTTGG |
| Cytochrome P450 CYP81A9 | GRMZM2G090432-RL | AGAAGTCCGAGCCAGAGG |
|  | GRMZM2G090432-RR | GGTGGTTCAGCAGCAGTG |
| Histone H2A | GRMZM2G003306-RL | GCTCCTGCCCAAGAAGAC |
|  | GRMZM2G003306-RR | CCAATAACAACGACGACACTAC |
| Hypothetical protein | GRMZM2G033799-RL | CTATCAACCTGCTAAACACACAAG |
|  | GRMZM2G033799-RR | GCTAAGTATCCAAGTCTCCAACC |
| Hypothetical protein | GRMZM2G122335-RL | ATGTCAGCCTATGCCTAC |
|  | GRMZM2G122335-RR | CATCTGTGATACTACCTTGG |
| No information | GRMZM2G154460-RL | CTTCCGTACCTCCTCCTCCTC |
|  | GRMZM2G154460-RR | CCCAGCCACCACCCACAG |
| Histone H2B.3 | GRMZM2G057852-RL | CCGTCACCAAGTTCACCTC |
|  | GRMZM2G057852-RR | CAGATCATATCATCCATCACTACC |
